# Combining chemotherapeutic agents and netrin-1 interference potentiates cancer cell death

**DOI:** 10.1002/emmm.201302654

**Published:** 2013-10-08

**Authors:** Andrea Paradisi, Marion Creveaux, Benjamin Gibert, Guillaume Devailly, Emeline Redoulez, David Neves, Elsa Cleyssac, Isabelle Treilleux, Christian Klein, Gerhard Niederfellner, Philippe A Cassier, Agnès Bernet, Patrick Mehlen

**Affiliations:** 1 Apoptosis, Cancer and Development Laboratory – Equipe labellisée ‘La Ligue’, LabEx DEVweCAN, Centre de Cancérologie de Lyon, INSERM U1052-CNRS UMR5286, Université de Lyon, Centre Léon BérardLyon, France; 2 Netris PharmaLyon, France; 3 Biopathology Department, Centre Léon BérardLyon, France; 4 Discovery Oncology Roche Diagnostics GmbHPenzberg, Germany

**Keywords:** cancer therapy, DCC, dependence receptor, netrin-1, p53

## Abstract

The secreted factor netrin-1 is upregulated in a fraction of human cancers as a mechanism to block apoptosis induced by netrin-1 dependence receptors DCC and UNC5H. Targeted therapies aiming to trigger tumour cell death via netrin-1/receptors interaction interference are under preclinical evaluation. We show here that Doxorubicin, 5-Fluorouracil, Paclitaxel and Cisplatin treatments trigger, in various human cancer cell lines, an increase of netrin-1 expression which is accompanied by netrin-1 receptors increase. This netrin-1 upregulation which appears to be p53-dependent is a survival mechanism as netrin-1 silencing by siRNA is associated with a potentiation of cancer cell death upon Doxorubicin treatment. We show that candidate drugs interfering with netrin-1/netrin-1 receptors interactions potentiate Doxorubicin, Cisplatin or 5-Fluorouracil-induced cancer cell death *in vitro*. Moreover, in a model of xenografted nude mice, we show that systemic Doxorubicin treatment triggers netrin-1 upregulation in the tumour but not in normal organs, enhancing and prolonging tumour growth inhibiting effect of a netrin-1 interfering drug. Together these data suggest that combining conventional chemotherapies with netrin-1 interference could be a promising therapeutic approach.

## INTRODUCTION

Netrin-1, a soluble protein initially discovered as an axon navigation cue (Serafini et al, 1994), was recently proposed to play a crucial role in cancer progression by regulating apoptosis (Mazelin et al, 2004; Mehlen et al, 2011). Indeed, netrin-1 receptors DCC and UNC5H,—*i.e*., UNC5H1, UNC5H2, UNC5H3 and UNC5H4 also called UNC5A, UNC5B, UNC5C or UNC5D—belong to the so-called dependence receptor family (Llambi et al, 2001; Mehlen et al, 1998; Tanikawa et al, 2003). These dependence receptors, because of their ability to induce cell death when disengaged from their ligands, create cellular states of dependence on their respective ligands (Bredesen et al, 2005) and, consequently, may behave as tumour suppressors as they eliminate tumour cells that would develop in settings of ligand unavailability (Mazelin et al, 2004; Mehlen & Puisieux, 2006). Along these lines, mice bearing a DCC receptor inactivated for its pro-apoptotic activity developed spontaneous colorectal cancers and were more prone to intestinal tumour progression (Castets et al, 2012). Similarly, inactivation of UNC5H3/C in mice in the gastro-intestinal tract is associated with intestinal tumour progression (Bernet et al, 2007).

Thus, according to the dependence receptor paradigm, progression of aggressive human tumours should require inactivation of this death pathway. There are at least three means to achieve this survival advantage: loss of netrin-1 receptors expression, as extensively described in human colorectal cancer for DCC or/and UNC5H (Bernet et al, 2007; Fearon et al, 1990; Shin et al, 2007; Thiebault et al, 2003); loss of downstream death signalling induced by DCC or UNC5H; or gain of autocrine or paracrine expression of the ligand. Interestingly, netrin-1 has been shown to be upregulated in a sizeable fraction of metastatic breast, lung, ovary and pancreatic cancer, in inflammatory-associated-colorectal cancer and in neuroblastoma (Delloye-Bourgeois et al, 2009a; Delloye-Bourgeois et al, 2009b; Dumartin et al, 2010; Fitamant et al, 2008; Papanastasiou et al, 2011; Paradisi et al, 2009). Proof-of concept studies, *in vitro* and in mice or chicken models, have shown that the silencing of netrin-1 by netrin-1 siRNA or interference with netrin-1–receptors interaction are associated with tumour cell death and with the inhibition of tumour growth and metastases (Delloye-Bourgeois et al, 2009a; Delloye-Bourgeois et al, 2009b; Dumartin et al, 2010; Fitamant et al, 2008; Paradisi et al, 2009). These later studies proposed that disrupting the netrin-1 binding to its receptors could represent an efficient anti-cancer strategy in the large fraction of cancers where netrin-1 is expressed in an autocrine or paracrine fashion. Early drug developments have focused on biological agents—biologics—that mimic receptors interaction with netrin-1 (Mille et al, 2009).

The search for the fraction of cancer patients who could be eligible for netrin-1 interference-based treatment during early clinical evaluation led us to examine the effects of conventional chemotherapeutic treatments on netrin-1 and netrin-1 receptors expression. Doxorubicin, 5-Fluorouracil (5FU), paclitaxel (Taxol) and Cisplatin are ‘classic’ chemotherapies that are still broadly used in the management of patients with breast, lung, colorectal, as well as other types of solid tumours; both in patients with localized and advanced tumours. However, despite their efficacy, the use of conventional agents is limited by their toxicity and the emergence of resistance. We show here that these chemotherapeutic treatments, even though they act via different cellular mechanisms, trigger a significant increase of netrin-1 and its receptors. We show that this upregulation is associated with an increased cell death induction upon inhibition of netrin-1 *in vitro*. As a consequence, we show that combination of Doxorubicin with a netrin-1 interfering drug candidate potentiates tumour growth inhibiting effect in an animal model.

## RESULTS

### Netrin-1 and its receptors are upregulated in tumour cells upon treatment with conventional chemotherapies

**Figure 1:**
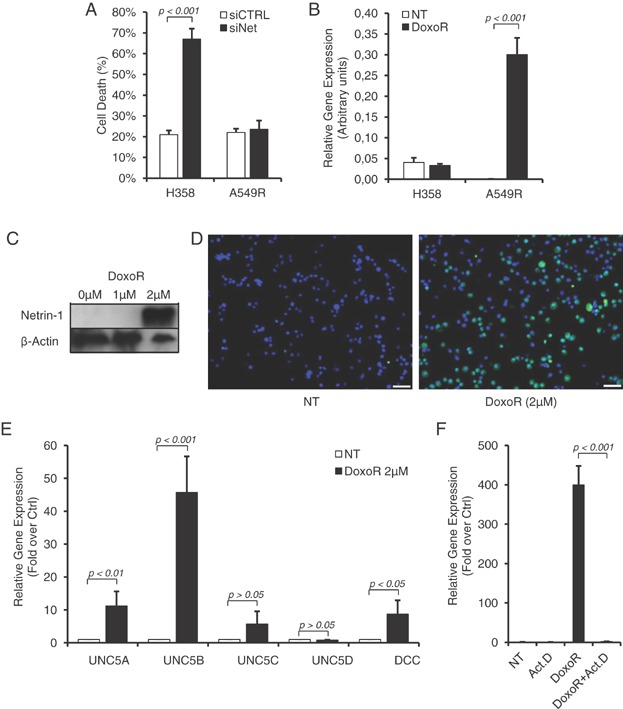
Netrin-1 and its dependence receptors are upregulated upon Doxorubicin treatment. Source data is available for this figure in the Supporting Information.

Based on the screen for netrin-1 expression in human tumour cell lines, we noticed that while a fraction of cell lines express netrin-1 and undergo apoptosis upon netrin-1 silencing [Fig 1A and (Delloye-Bourgeois et al, 2009a)], other cell lines failed to show netrin-1 expression and are resistant to netrin-1 interference (Fig 1A). Of interest, we observed that Doxorubicin treatment, while having no effect on netrin-1 expressing H358 cells, is associated with netrin-1 over-expression in a Doxorubicin resistant lung cancer cell line A549R (Fig 1B). This increase of mRNA was associated with a robust increase of netrin-1 protein expression (Fig 1C and D).

1. Lung cancer cell lines H358 and A549R were transfected with a scramble siRNA (siCTRL) or a netrin-1 targeting siRNA (siNet). 48 h after transfection, cell death percentage (*n* = 3) was evaluated by means of 4′,6-diamidino-2-phenylindole (DAPI) exclusion.
2. Netrin-1 gene expression, normalized with Glyceraldehyde 3-phosphate dehydrogenase (GAPDH) and expressed as arbitrary units, was evaluated in H358 and A549R cell lines, treated with 2 µM Doxorubicin for 24 h (DoxoR) or not treated (NT). Results represent mean values of five independent experiments.
3. A549R cells were treated with 1 and 2 µM Doxorubicin for 48 h and netrin-1 protein levels were evaluated by western blotting. Netrin-1 protein, normalized to β-actin, was strongly accumulated following Doxorubicin treatment.
4. Netrin-1 (in green) upregulation was confirmed by immunofluorescence staining, following treatment with 2 µM Doxorubicin for 48 h. Nuclei were counterstained with Hoescht staining (in blue). White scale bar: 40 µm.
5. Netrin-1 receptors gene expression was measured in A549R cells following Doxorubicin treatment. UNC5A, UNC5B and DCC gene expression was significantly upregulated by Doxorubicin, while UNC5C and UNC5D showed non-significant variations.
6. Doxorubicin-induced netrin-1 upregulation is directly dependent by gene transcription. A549R cells were treated with 2 µM Doxorubicin and with the potent RNA polymerases inhibitor Actinomycin D (100 µg/ml) for 24 h. Actinomycin D strongly inhibited netrin-1 upregulation following Doxorubicin treatment. In (A), (B), (E) and (F), Mann–Whitney tests were performed, and *p*-value is indicated. DoxoR, Doxorubicin; Act.D, Actinomycin D; NT, not treated.

Next, we analysed the level of the netrin-1 receptors DCC and UNC5H—UNC5A, UNC5B, UNC5C and UNC5D—in response to Doxorubicin. As shown in Fig 1E, levels of DCC, UNC5A, UNC5B and UNC5D increase concomitantly to netrin-1 levels, in A549R cells treated with Doxorubicin. This increase reached 44-folds for the UNC5B receptor. To monitor whether this upregulation of netrin-1 and its receptors is related to increased gene transcription, A549R cells were treated with Doxorubicin in the presence of the RNA polymerase inhibitor Actinomycin D. As shown in Fig 1F, Actinomycin D fully prevents Doxorubicin-mediated netrin-1 upregulation, thus supporting the view that conventional therapeutic drugs triggers increase of netrin-1 and its receptors via enhanced gene transcription. This increased gene transcription is associated with increased receptor presence at the plasma membrane as shown with the receptor DCC by flow cytometry analysis (Supporting Information Fig S1A).

**Figure 2:**
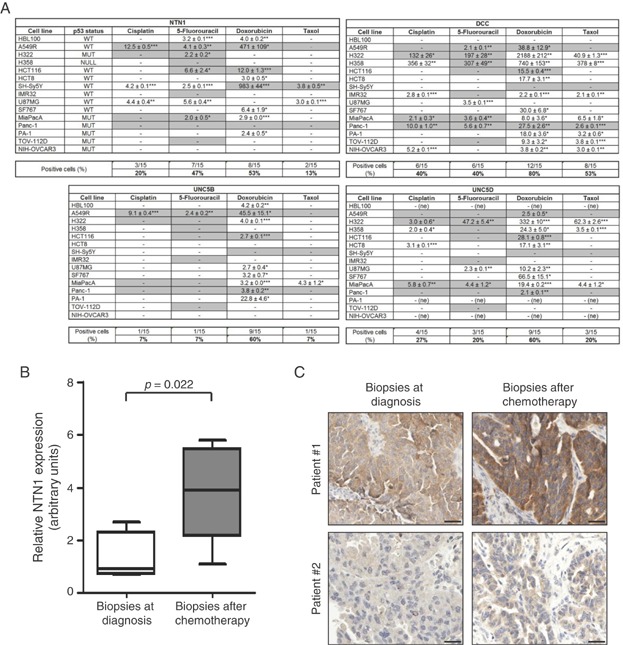
Netrin-1 and its receptors expression is increased in several cancer cell lines and in ovarian tumours upon treatment with cytotoxic drugs.

To investigate whether upregulation of netrin-1 and its receptors is restricted to Doxorubicin or is a general response to chemotherapeutic agents, netrin-1 levels were analysed by quantitative RT-PCR in a panel of 15 cancer cell lines in response to various conventional chemotherapeutic drugs, such as Doxorubicin, 5-Fluoruracil (5FU), paclitaxel (Taxol) and Cisplatin. Analysis of netrin-1 level was performed upon treatment with 3 concentrations corresponding to the determined IC_10_, IC_30_ or IC_50_ of each drug for each cell lines (Supporting Information Table S1). In cell lines which appeared to be resistant to specific drugs (Supporting Information Table S1), a concentration corresponding to maximal effective concentration (IC_MAX_) was used to monitor netrin-1 level. As shown in Fig 2A, Doxorubicin and 5FU both triggered a significant (*i.e*. >2-fold over control) increase of netrin-1 respectively in 53 and 47% of cancer cell lines. Treatment with Taxol and Cisplatin was associated with netrin-1 upregulation, respectively, only in 13 and 20% of cell lines. Netrin-1 upregulation upon chemotherapeutic drugs treatment is not tumour type specific as netrin-1 upregulation was seen in at least one cell line of breast, lung, colon, pancreatic and ovarian cancers, as well as in neuroblastoma and glioblastoma cell lines. We could not detect any correlation between netrin-1 upregulation and chemoresistance, as netrin-1 upregulation was detected in both resistant and sensitive cell lines, and as some resistant cell lines did not show netrin-1 upregulation (Fig 2A).

1. Expression levels of netrin-1 (NTN1), DCC, UNC5B and UNC5D were measured by quantitative RT-PCR. Breast cancer (HBL100), lung cancer (A549R, H322, H358), colon cancer (HCT116, HCT8), pancreatic cancer (MiaPacA-2, Panc-1), neuroblastoma (SH-Sy5y, IMR32), glioblastoma (SF767, U87MG) and ovarian cancer (PA-1, TOV-112D, NIH-OVCAR3) cell lines were treated with classical chemotherapeutic drugs (Doxorubicin, Cisplatin, 5-Fluorouracil and Taxol), at different drugs concentration dependent on IC_50_ values calculated for each cell line and drug treatment for 24 h. Netrin-1 and its receptors gene expression was compared to control, not-treated cells, values indicate fold over control changes. Standard deviation and statistical analysis are also indicated. –, no changes or downregulation; n.e., not expressed. Positive cell lines were determined for gene expression variations more than twofold over control. Grey boxes represent resistant (*i.e*. more than 50% cell survival after treatment with maximal drugs concentrations) cancer cell lines. p53 status is indicated for each cell line (WT, wild-type p53; MUT, mutated p53; NULL, deleted p53).
2. Netrin-1 is over-expressed in ovarian tumour patients after chemotherapeutic treatment. Netrin-1 level, normalized with glyceraldehyde 3-phosphate dehydrogenase (GAPDH) gene, used as housekeeping gene, was analysed in RNA extracted from ovarian biopsies of tumours from patients obtained before and after a chemotherapeutic cycle of carboplatin/taxol treatment (*n* = 5 for each group). The median level of netrin-1 was calculated for each group. Mann–Whitney test was performed, and *p*-value is indicated.
3. Immunohistochemistry staining of two representive ovarian cancer biopsies, obtained before and after the conventional treatment. Original magnification, ×20. Scale bar: 50 µm. After chemotherapy, a high (patient 1) or intermediate (patient 2) intensity of the cytoplasmic staining indicated higher levels of netrin-1 as compared to staining obtained before treatment.

The expression of netrin-1 dependence receptors in response to these cytotoxic agents was also investigated in the 15 cancer cell lines (Fig 2A). Similarly to netrin-1 response, Doxorubicin seemed to have the largest effect, as it is associated with the upregulation of DCC, UNC5B and UNC5D, respectively, in 80, 60 and 60% of the cell lines screened. DCC, which displays an overall low expression in the screened cancer cell lines, is the netrin-1 receptor showing the largest spectrum of upregulation as DCC expression was strongly increased in 40, 40 and 53% of cell lines in response to, respectively, Cisplatin, 5FU and Taxol. Levels of the netrin-1 receptors UNC5A and UNC5C remained largely unaffected by the treatment with cytotoxic drugs in most of the cell lines that were screened (Supporting Information Fig S1B). Collectively, these data support the view that upregulation of netrin-1 and its receptors frequently occurs in response to conventional drug treatment.

To further analyse whether conventional drug treatments trigger tumour-specific increased netrin-1 level in human patients, we looked for pathologies where human tumours are sampled from the same patient before and after conventional treatments. We were then able to analyse netrin-1 and receptors level in ovarian cancer specimens from patients before and after being treated with carboplatin/taxol. As shown in Fig 2B, netrin-1 mRNA was significantly upregulated after chemotherapy. Moreover, DCC, UNC5B, UNC5C and UNC5D levels were also affected by the carboplatin/taxol treatment (Supporting Information Fig S1C–F). Netrin-1 upregulation was also confirmed at the protein level; by netrin-1 immunohistochemistry in tissue sections derived these biopsies (Fig 2C). Increased netrin-1 intensity was detected in 3 out of 4 analysed biopsies after chemotherapy.

### Netrin-1 interference potentiates cytotoxic drugs induced cell death and tumour growth inhibition

**Figure 3:**
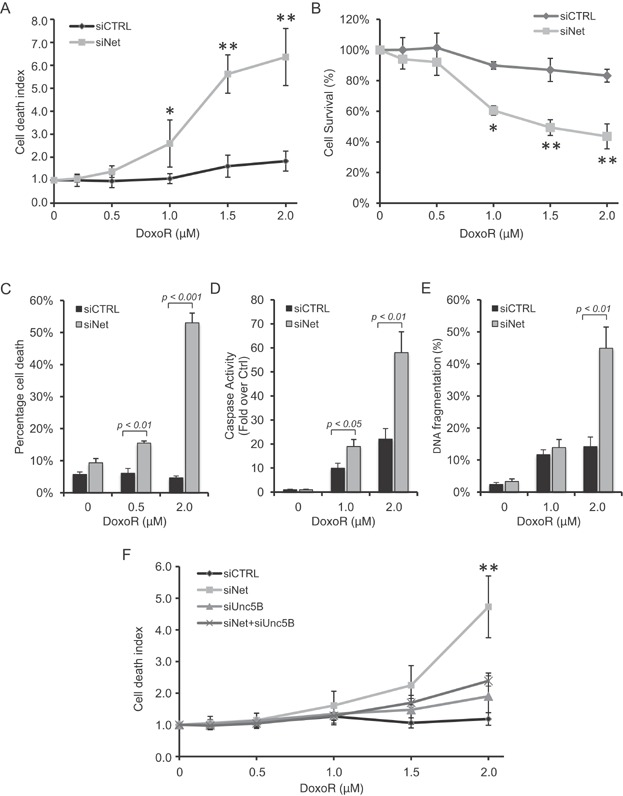
Netrin-1 silencing sensitizes A549R cells to Doxorubicin and induces apoptotic cell death via UNC5B receptor.

The fact that both netrin-1 and its receptors are upregulated upon conventional drug treatments suggests that the dependence for survival on netrin-1 is amplified in chemotherapy-treated cancer cells. Thus, we analysed the effect of silencing netrin-1 by siRNA on Doxorubicin-induced cell death. A549R cells were transfected with a netrin-1 siRNA and treated with increasing concentration of Doxorubicin. Silencing of netrin-1 (Supporting Information Fig S2AB) was associated with a marked potentiation of Doxorubicin-induced cell death as shown by the measurement of loss of cell permeability (Fig 3A), cell survival (Fig 3B), DAPI exclusion (Fig 3C), caspase activation (Fig 3D) or DNA fragmentation (Fig 3E). To determine whether the increased sensitivity was due to the pro-apoptotic engagement of unbound netrin-1 dependence receptors, a similar experiment was performed in settings of silencing of UNC5B (Supporting Information Fig S2AB), the main netrin-1 receptor expressed upon Doxorubicin treatment in A549R cells. As shown in Fig 3F, silencing of UNC5B is associated with the inhibition of the potentiation of cell death induced by netrin-1 silencing and Doxorubicin treatment.

A–C. Netrin-1 silencing sensitizes tumour cells to Doxorubicin. A549R cells were transfected with either a scramble siRNA (siCTRL) or with a specific siRNA targeting netrin-1 (siNet). 24 h after transfection, cells were treated with increasing concentrations of Doxorubicin. Cell death rate (A), measured by toxilight kit as described in materials and methods section, and cell survival (B), was evaluated 48 h after treatment. Results were normalized to control, untreated cells. While scramble siRNA-transfected cells showed a general resistance to Doxorubicin treatment, netrin-1 silencing strongly induced cell death and decreased cell survival in presence of Doxorubicin. Evaluation of cell death percentage (C), measured by 4′,6-diamidino-2-phenylindole (DAPI) exclusion as described in the materials and methods section, confirmed that netrin-1 siRNA sensitized A549R cells to 0.5 and 2 µM Doxorubicin treatment.
D,E. Netrin-1 silencing triggers apoptosis in combination with Doxorubicin treatment. A549R cells were transfected as in (A–C), and treated with the indicated Doxorubicin concentrations for 24 h. Active caspase-3 (D), normalized to untreated cells, and DNA fragmentation (E) were evaluated as described in the materials and methods section. While Doxorubicin failed to induce apoptosis in A549R cells transfected with a scramble siRNA (siCTRL), cells silenced for netrin-1 showed a strong increase in the apoptotic rate.
F. Combination of netrin-1 silencing and Doxorubicin treatment induces cell death through netrin-1 receptor UNC5B. A549R cells were transfected with scramble siRNA (siCTRL), netrin-1-specific siRNA (siNet), UNC5B-specific siRNA (siUnc5B) and with a combination of netrin-1 and UNC5B-targeting siRNA. 24 h after transfection, cells were treated with the indicated Doxorubicin concentrations for 48 h, and cell death rate was measured by toxilight and normalized to control, untreated cells. While netrin-1 silencing (siNet) sensitized A549R cells to Doxorubicin treatment, as compared to siCTRL-transfected cells, the simultaneously silencing of netrin-1 and UNC5B (siNet + siUnc5B) rescued cell death induction by siNet and Doxorubicin treatment. *, *p* < 0.05; **, *p* < 0.01. DoxoR, Doxorubicin.

**Figure 4:**
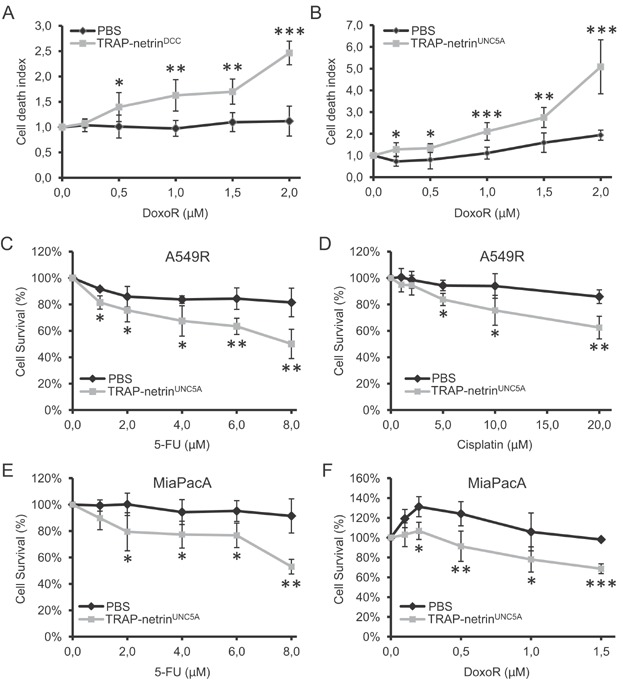
Interference to netrin-1 and its receptors interaction sensitizes tumour cells to cytotoxic drugs.

We thus investigated a possible similar potentiating effect using a more therapeutically relevant way for interfering with netrin-1. Two drug candidates, TRAP-netrin^DCC^ and TRAP-netrin^UNC5A^, which are Fc-fused and stabilized ectodomains of respectively DCC or UNC5A, have been shown to trigger death of netrin-1 expressing tumour cells *in vitro* and tumour growth inhibition in engrafted mice models (not shown). As shown in Fig 4AB, these two candidate drugs strongly potentiate Doxorubicin-induced cell death in A549R cells. Moreover, we confirmed that co-treatment with TRAP-netrin^UNC5A^ and Doxorubicin induced DAPK dephosphorylation (Supporting Information Fig S2C), an event associated with cell death induced by unbound UNC5B receptor (Llambi et al, 2005).

A,B. A549R cells were treated for 48 h with the indicated Doxorubicin concentrations in presence or not of 2 µg/ml TRAP-netrin^DCC^ (A) and TRAP-netrin^Unc5A^ (B). The co-treatment with Doxorubicin and the two recombinant fusion proteins increased cell death rate, measured by toxilight, as compared to Doxorubicin- and PBS-treated cells. Results were normalized to untreated cells.
C,D A549R cells were treated with PBS or 2 µg/ml TRAP-netrin^Unc5A^, in presence of the indicated concentrations of 5-Fluorouracil (5-FU, C) or Cisplatin (D). 48 h after co-treatment, cell survival was measured by MTS and normalized to untreated cells.
E,F. MiaPacA cells were treated with PBS or 2 µg/ml TRAP-netrin^Unc5A^, in presence of the indicated concentrations of 5-FU (E) or Doxorubicin (F). 48 h after co-treatment, cell survival was measured by MTS and normalized to untreated cells.

As netrin-1 and receptors were also upregulated upon 5FU and Cisplatin treatment (Fig 2A), we performed similar combination of TRAP-netrin^UNC5A^ with 5FU and Cisplatin. A comparable potentiating effect on cell death was observed upon co-treatment with 5FU or Cisplatin and TRAP-netrin^UNC5A^ (Fig 4CD). Similarly, in pancreatic cancer cell line MiaPacA, where 5FU and Doxorubicin have been shown to upregulate netrin-1 and its receptors, co-treatment of 5FU or Doxorubicin and TRAP-netrin^UNC5A^ potentiated cell death (Fig 4EF).

**Figure 5:**
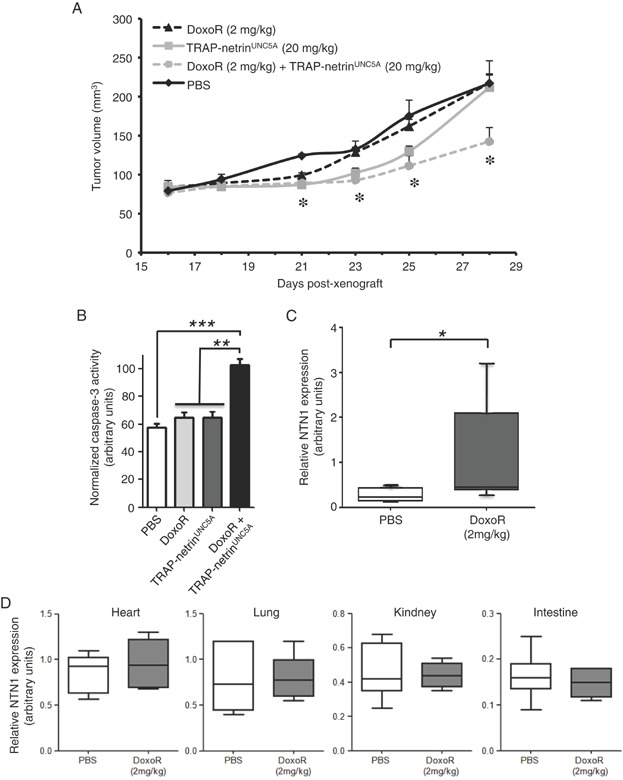
Tumour growth inhibiting effect of combining netrin-1 interference and Doxorubicin.

We then assessed whether the *in vitro* effect seen above could be translated *in vivo* in a therapeutic setting. A549R cells were engrafted in *nude* mice and animals with established palpable tumours were treated twice a week by i.p. injection of vehicle or TRAP-netrin^UNC5A^ at 20 mg/kg alone, or in combination with 2 mg/kg of Doxorubicin. Single agent treatment (TRAP-netrin^UNC5A^ or Doxorubicin) according to this administration scheme and doses were associated with detectable but weak tumour growth inhibiting effect, which was resolved during the time of the treatment (Fig 5A). However, co-treatment of Doxorubicin and TRAP-netrin^UNC5A^ was associated with a stronger and prolonged inhibition of tumour growth. The stronger and prolonged effect is associated with increased tumour apoptosis. Indeed, we assessed apoptosis level in the xenografts tumours after 48 h of treatment with either Doxorubicin, TRAP-netrin^UNC5A^ or the combination of both agents. As shown in Fig 5B, while Doxorubicin or TRAP-netrin^UNC5A^ alone failed to significantly induce caspase-3 activity in the tumours, the combination triggers a strongly significant caspase-3 activation. Of interest, Doxorubicin treatment (i.p.) is associated with upregulation of netrin-1 in the xenograted tumours (Fig 5C) but not in tissues such as the heart, lung, intestine or kidney (Fig 5D). Together, these data support the view that Doxorubicin triggers netrin-1 upregulation specifically in tumours and not in normal tissues; an effect that can be used to potentiate the anti-tumour effect of netrin-1 interfering agents.

1. TRAP-netrin^Unc5A^ potentiates Doxorubicin anti-cancer effect in a preclinical animal model. A549R cells were engrafted in 7-weeks old female athymic nude mice. Once tumours reached a 100 mm^3^-volume, mice were treated intraperitoneally with TRAP-netrin^UNC5A^ (20 mg/kg), Doxorubicin (2 mg/kg) or with a combination of both drugs, twice a week for two weeks. As a control, mice were injected with PBS. Histogram represents tumour volume growth for each group as a function of days post-xenografts. While both drugs alone were not able to reduce tumour growth, combination of TRAP-netrin^UNC5A^ and Doxorubicin treatment significantly reduced tumour growth.
2. Quantification of apoptosis by caspase-3 activity assay on xenografts lysates analysed after 2 days of treatment. Data are means of caspase-3 activities in four xenograft tumour lysates for each condition.
3. Quantification of netrin-1 expression on engrafted A549R cells from *nude* mice treated with PBS or Doxorubicin for 48 h. Netrin-1 expression was normalized using hypoxanthine-guanine phosphoribosyltransferase (HPRT) and TATA binding protein (TBP) genes, used as housekeeping genes. Data are means of total RNA extracted from seven animals for each group. Median, min and max values, as well as the upper and lower quartiles, are indicated.
4. Quantification of netrin-1 expression in non-tumoural tissues from *nude* mice treated as above. The indicated tissues were taken 48 h after treatment and total RNA was extracted. Netrin-1 expression was normalized using 60S acidic ribosomal protein P2 (RPLP2) gene, used as housekeeping gene. *, *p* < 0.05; **, *p* < 0.01; ***, *p* < 0.001; DoxoR, Doxorubicin.

### Netrin-1 upregulation upon cytotoxic drugs is mediated by p53

**Figure 6:**
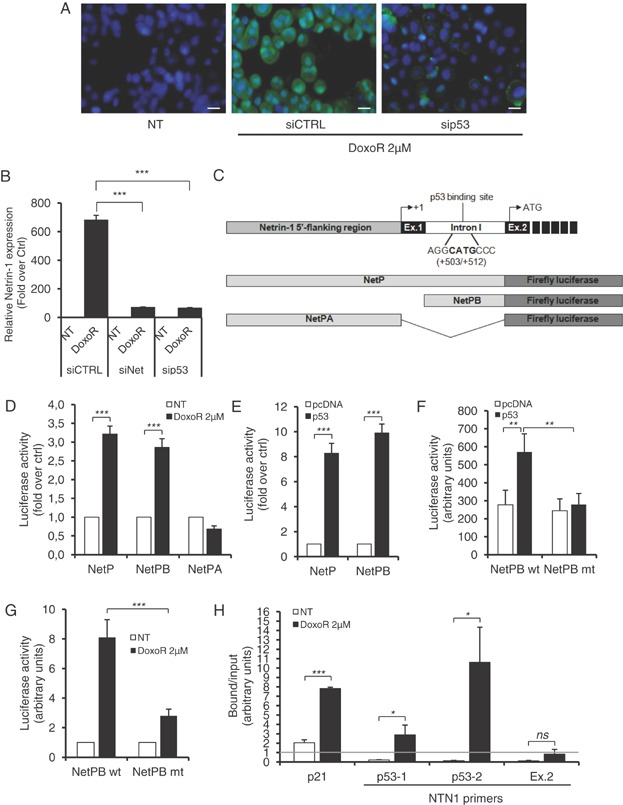
Netrin-1 upregulation is p53-dependent.

The netrin-1 upregulation, observed in various cell lines and tumours with different cytotoxic drugs, suggests that this upregulation is rather related to a general survival stress response than to a specific alteration of a specific pathway. Since cytotoxic drugs often activates the tumour suppressor p53 (Vousden & Lane, 2007) and because it was shown that UNC5A, UNCB and UNC5D are transcriptional target of p53 (Miyamoto et al, 2010; Tanikawa et al, 2003; Wang et al, 2008), we investigated whether p53 could be implicated in Doxorubicin-induced netrin-1 over-expression. A549R cells treated with Doxorubicin showed accumulation and activation of p53 (Supporting Information Fig S3ABCD). Interestingly, while netrin-1 mRNA and protein were upregulated upon Doxorubicin treatment, this upregulation was strongly inhibited when p53 was silenced by a siRNA strategy (Fig 6AB). Analysis of the netrin-1 promoter revealed the presence of a putative p53 binding site in the internal netrin-1 promoter [Fig 6C; netrin-1 promoter B (Delloye-Bourgeois et al, 2012)]. As shown in Fig 6DE, Doxorubicin treatment or p53 over-expression similarly trigger transcription of a luciferase reporter gene placed under the control of the complete netrin-1 promoter region or of the netrin-1 promoter B but not of netrin-1 promoter A region (Paradisi et al, 2008). Mutation of the p53 binding site in promoter B fully abrogates p53 or Doxorubicin-induced promoter activation (Fig 6FG). Moreover, by using chromatin-immunoprecipitation assay, we demonstrated that upon Doxorubicin treatment, p53 specifically interacts with netrin-1 promoter B (Fig 6H). Of interest, and although it remains to be further demonstrated on a larger panel of human tumours, we found a significant correlation between netrin-1 level before and after chemotherapy and p53 status (monitored by measuring p53 targets) in the human specimen described in Fig 2B (Supporting Information Fig S3EFG). The direct contribution of p53 in Doxorubicin-dependent-netrin-1 upregulation was further analysed in colorectal cancer cell line HCT116 either expressing wild-type p53 (p53^+/+^) or without p53 (p53^−/−^) (Bunz et al, 1998). While HCT116 p53^+/+^ cells showed a strong induction of netrin-1 and p21 gene expression following Doxorubicin treatment, we failed to observe netrin-1 upregulation in HCT116 p53^−/−^ cells (Supporting Information Fig S4AB). As expected, p53^+/+^ cells were sensitive to co-treatment with Doxorubicin and TRAP-netrin^UNC5A^, while p53^−/−^ cells were not (Supporting Information Fig S4C).

A. A549R cells were transfected with p53 siRNA (sip53) or control siRNA (siCTRL) and treated with 2 µM Doxorubicin for 48 h. Endogenous netrin-1 protein (green) was detected by immunofluorescence staining. Nuclei were counterstained with Hoescht staining (in blue). While Doxorubicin strongly increased netrin-1 protein, p53 silencing was able to revert this upregulation. White scale bar: 20 µm.
B. Netrin-1 expression levels in p53- and netrin-1-silenced A549R cells, treated with 2 µM Doxorubicin for 24 h, were evaluated by quantitative RT-PCR.
C. Schematic representation of the 5′-flanking netrin-1 genomic region. The 5′-flanking region, containing the netrin-1 promoter A (grey box) (Paradisi et al, 2008), the first two exons (solid box), the first intron (open box), containing the alternative promoter B, described in(Delloye-Bourgeois et al, 2012), the transcription start site +1, and the putative p53 binding site, are indicated. The different netrin-1 promoter A (NetPA) and B (NetPB) region, and the full promoter region (NetP) were cloned upstream the firefly luciferase gene.
D. A549R cells were transfected with the indicated netrin-1 promoter constructs and treated with 2 µM Doxorubicin for 24 h. Netrin-1 promoter activity was then evaluated by measuring luciferase activity, as indicated in the materials and methods section.
E. A549R cells were co-transfected with the netrin-1 promoter constructs NetP and NetPB and p53, and luciferase activity was measured 48 h later. Over-expression of p53 was able to trans-activate both the promoter constructs.
F,G. Mutation of the putative p53 binding site abolished netrin-1 promoter trans-activation by Doxorubicin or p53 over-expression. A549R cells were transfected with NetPB promoter construct wild-type (NetPB wt) or mutated for the p53 binding site (NetPB mut). While treatment with Doxorubicin (F) or p53 over-expression (G) was able to trans-activate the wild-type netrin-1 promoter, abolishing p53 binding was sufficient to decrease netrin-1 promoter activation.
H. Chromatin Immunoprecipitation (ChIP) assay was performed in A549R cells treated with 2 µM Doxorubicin for 24 h, as described in the material and methods section. p53-co-precipitated DNA was analysed by quantitative PCR. Two different couples of primers, surrounding the p53 binding site, were used for netrin-1 (p53-1 and p53-2). A couple of primers, able to amplify a DNA fragments corresponding to netrin-1 exon 2 (Ex.2), were used as negative control. p21 primers, surrounding the p53 binding site -1354 contained in the p21 promoter(Macleod et al, 1995), were used as positive control for p53 activation and binding. While in control, not treated cells p53 was not able to bind netrin-1 genomic DNA, in Doxorubicin-treated cells we observed a strong enrichment of netrin-1 DNA surrounding the p53 binding site, but not in the netrin-1 DNA corresponding to exon 2, in the p53 co-immunoprecipitate. Grey lane represents the threshold of enriched DNA region that co-immunoprecipitate with p53. *, *p* < 0.05; **, *p* < 0.01; ***, *p* < 0.001; *ns*, not significant; NT, not treated; DoxoR, Doxorubicin.

Some of the p53 mutations are not loss of wild-type p53 function, but they contribute to creating mutant p53 isoforms that possess protumoural activities. These p53 mutations are known as ‘gain-of-functions’ (GOF) mutations and they are selected by cancer cells to overcome p53 anti-apoptotic activity. To test the effect of GOF mutant p53 proteins on netrin-1 expression, we transfected HCT116 p53^−/−^ cells with such mutant isoforms. As shown in Supporting Information Fig S4D, only wild-type p53 is able to activate netrin-1 promoter, while the large majority of GOF mutants showed no effect on netrin-1 promoter activation. As p53 is not only activated by conventional chemotherapeutic treatment, we finally investigated whether other p53 inducer may also induce netrin-1 expression. As shown in Supporting Information Fig S4EF, treatment of A549R cells with hydrogen peroxide (H_2_O_2_), known to induce oxidative stress and p53 activation, is sufficient to stimulate netrin-1 expression. All together, these data suggest that in p53 non-mutated cancer cells, netrin-1 is upregulated via p53 activation and p53-mediated netrin-1 promoter activation.

## DISCUSSION

Previous works have shown that a sizeable fraction of human cancer showed upregulation of netrin-1 as a selective mechanism to block tumour cell death (Delloye-Bourgeois et al, 2009a; Delloye-Bourgeois et al, 2009b; Fitamant et al, 2008; Paradisi et al, 2009). This acquired selective advantage is in a sense very similar to the mechanism of oncogene addiction. Of interest, we describe here that the fraction of cancer that could show dependence receptor dependent apoptosis inhibition, may even be larger as many tumours treated with conventional chemotherapies may potentially upregulate netrin-1. The cytotoxic drugs tested here, which included Doxorubicin, Cisplatin, 5FU and paclitaxel (Taxol) are commonly used in the management of patients with non-small cell lung, breast, colorectal and ovarian cancers; both in the adjuvant and advanced setting. Moreover, we have shown using a panel of human samples that primary ovarian tumours from patients treated with Carboplatin/Taxol displays an increase in netrin-1 level compared to the same tumours before treatment. Although, netrin-1 upregulation in cell culture differs in kinetics and amplitude, depending on the drug used and the cancer cell type (Fig 2A), the fact that these drugs are known to affect different cellular mechanisms supports the view that netrin-1 upregulation is rather a general survival stress response than a specific alteration of a specific pathway affected by a specific chemotherapeutic drug. It is therefore interesting to speculate that the netrin-1 upregulation may be a survival mechanism employed by cancer cells in response to these drugs.

Although the mechanisms for this upregulation of netrin-1 remain to be further investigated, we propose here that, at least in cancer cells in which p53 is not affected, this upregulation may be due to p53-mediated activation of netrin-1 promoter. It is however fair to say that probably other mechanisms may be implicated in netrin-1/receptors upregulation. With respect to this, NF-κB also regulates netrin-1 transcription (Paradisi et al, 2008; Paradisi et al, 2009) and it has been shown that Doxorubicin chemoresistance could be linked to NF-κB activation (Mi et al, 2008). However, inhibition of NF-κB is not sufficient to inhibit Doxorubicin-dependent netrin-1 upregulation, at least in A549R cell line (data not shown). If the mechanisms for netrin-1 upregulation are probably more complex, it may have significant therapeutic consequences. Indeed, netrin-1 interfering drugs are currently under preclinical development; combination of these compounds with conventional cytotoxic agents may prove synergistic. We and others have shown that netrin-1 expression is upregulated in samples from breast, ovarian, pancreatic and non-small cell lung cancer patients and that interfering with the netrin-1 autocrine/paracrine loop triggers apoptosis of cancer cells in several models. Furthermore, the data presented here suggests that large subset of patients may benefit from netrin-1 targeting agents, either alone or in combination with cytotoxic agents. Based on our *in vivo* observation on tumour bearing mice, the combination mediates superior anti-tumoural efficacy, but does not appear to increase toxicity compared to cytotoxic agents alone. The pre-clinical data shown here support the view that combining conventional drugs with netrin-1 interference could lead to superior efficacy as well as a reduction of chemotherapy doses at increased efficacy. Together, these data support the rationale of testing netrin-1 interference based therapy in early clinical trials in combination with conventional chemotherapies.

## MATERIALS AND METHODS

### Quantitative RT-PCR

Total RNAs from cancer cell lines were extracted using NucleoSpin® RNA II Kit (Macherey Nagel, Düren, Germany) according to manufacturer's protocol. Total RNAs from mouse non-tumoural tissues, xenograft tumours and human ovarian tumours were extracted by disrupting tissues with MagNA Lyser instrument (Roche Applied Science). RT-PCR reactions were performed with iScript® cDNA Synthesis Kit (Biorad). One microgram total RNA was reverse-transcribed using the following program: 25°C for 5 min, 42°C for 30 min and 85°C for 5 min. For expression studies, the target transcripts were amplified in LightCycler® 2.0 apparatus (Roche Applied Science), using the LightCycler FastStart DNA Master SYBR Green I Kit (Roche Applied Science). Expression of target genes was normalized to glyceraldehyde 3-phosphate dehydrogenase (GAPDH), hypoxanthine-guanine phosphoribosyltransferase (HPRT), TATA binding protein (TBP) and phosphoglycerate kinase (PGK) genes, used as housekeeping genes. The amount of target transcripts, normalized to the housekeeping gene, was calculated using the comparative C_T_ method. A validation experiment was performed, in order to demonstrate that efficiencies of target and housekeeping genes were approximately equal. The sequences of the primers are available upon request.

### Netrin-1, DCC, p53 and p21 protein quantification in human cancer cells

For immunobloting analysis, cells were lysed by sonication in modified RIPA buffer (50 mM Tris-HCl, pH7.5, 150 mM NaCl, 1% NP-40, 0.5% sodium deoxycholate, 0.1% SDS, 1 mM EDTA, protease inhibitor cocktail and 5 mM DTT) and incubated 1 h at 4°C. Cellular debris were pelletted by centrifugation (10.000 *g* 15′ at 4°C) and protein extracts (200 μg per lane) were loaded onto 10% SDS-polyacrylamide gels and blotted onto PVDF sheets (Millipore Corporation, Billerica, MA, USA). Filters were blocked with 10% non-fat dried milk and 5% BSA in PBS/0.1% Tween 20 (PBS-T) over-night and then incubated for 2 h with rabbit polyclonal α-netrin-1 (dilution 1:500, clone H104, Santa Cruz Biotechnology, Santa Cruz, CA, USA), mouse monoclonal α-p53 (1:1000, clone DO-1, Santa Cruz Biotechnologies), rabbit polyclonal α-p21 (1:1000, clone C-19, Santa Cruz Biotechnologies) and mouse monoclonal α-actin (1:1000, Santa Cruz Biotechnologies) antibodies. After three washes with PBS-T, filters were incubated with the appropriate HRP-conjugated secondary antibody (1:10000, Jackson ImmunoResearch, Suffolk, UK) for 1 h. Detection was performed using West Dura Chemiluminescence System (Pierce, Rockford, IL, USA).

For immunofluorescence study, cells were detached, centrifuged on cover slips with a cytospiner (Shandon Cytospin 3, Thermo Scientific) and fixed for 30 min with 4% v/v paraformaldehyde. Cells were then permeabilized for 30 min in 0.2% Triton X-100/PBS and blocked in PBS containing 2% BSA and 2% normal donkey serum. Endogenous netrin-1 was stained using rat monoclonal α-netrin-1 antibody (R&D systems) and Alexa-488 Donkey anti-rat IgG (Molecular probes). p53 protein was stained using the mouse monoclonal α-p53-DO-1 antibody. Nuclei were counterstained using Hoescht staining (Sigma–Aldrich).

For DCC flow cytometry analysis, A549R cells were treated for 48 h with 2 µM Doxorubicin and after detachment cells were stained with α-DCC antibody (AF5, Abcam, Cambridge, MA, USA) diluted at 1/500 for 2 h at 4°C, following incubation for 1 h with phycoerytrin-conjugated anti-mouse antibody, diluted at 1/1000.

### Cell death assay and conventional drug treatments

Cell death was evaluated by means of different methods. For total cell death assays, 5 × 10^3^ cells per well were grown in 96-well plate in serum-poor medium and treated with Doxorubicin. 48 h later, cell death was evaluated using the bioluminescent cytotoxicity assay ToxiLight (Lonza, Basel, Switzerland), according to manufacturer's instruction. Alternatively, cell death percentage was measured by acridine orange and DAPI staining, using the NucleoCounter NC-3000 system (ChemoMetec A/S, Allerød, Denmark). Briefly, 5 × 10^4^ cells were plated in 12-well plate and treated with Doxorubicin. 48 h after treatment, floating and adherent cells were collected, suspended in PBS and mixed with two different dyes, acridine orange, staining the entire population of cells, and 4′,6-diamidino-2-phenylindole (DAPI), staining the non-viable cells. Cell death rate, measured as DAPI-positive cells in total cell population, was then determined by NucleoCounter NC-3000, following the manufacture's application note. Cell survival was measured by MTS assay (CellTiter 96 AQueous One Solution Cell Proliferation Assay, Promega) in 96-well plates. MTS assay was performed according to the manufacturer's procedures on 3 × 10^3^ cells grown in serum-poor medium for 16 h and then treated for 48 h with the indicated Doxorubicin concentrations in serum-free medium. Caspase-3 activity assay was performed as previously described (Paradisi et al, 2008) using the Caspase 3/CPP32 Fluorimetric Assay Kit (Gentaur Biovision, Brussel, Belgium), according to the manufacturer's instructions. Caspase activity (activity/min/microgramme of protein) was calculated from a 1 h kinetic cycle reading on a spectrofluorimeter (405 nm/510 nm, Infinite F500, Tecan, Männedorf, Switzerland).

### Candidate drugs

TRAP-netrin^DCC^ and TRAP-netrin^UNC5A^ are, respectively, the fifth fibronectin domain of DCC ectodomain and the Ig1–Ig2 domains of the UNC5A ectodomain fused to IgG1 Fc portion. These two recombinant proteins, described in, respectively, patent applications PCT/EP2011/064733 and EU/12306099.8 were produced, respectively, in 293-free-style and CHO-free-style and were provided, respectively, by Roche and Netris Pharma.

### Animal model

Seven-week-old (20–22 g body weight) female athymic nu/nu mice were obtained from Charles River animal facility. The mice were housed in sterilized filter-topped cages and maintained in a pathogen-free animal facility. A549R cells were implanted by s.c. injection of 10^7^ cells in 200 µl of PBS into the right flank of the mice. Once tumours were established (*V* ≈ 100 mm^3^), mice were treated with netrin-1 interfering drugs and/or cytotoxic drugs for 2 weeks. Tumour sizes were measured with a caliper. The tumour volume was calculated with the formula *v* = 0.5 × (length × width^2^). Four animals for each group were sacrificed 48 h after initial treatment, in order to evaluate caspase-3 activation in tumoural tissue and to quantify netrin-1 expression in engrafted tumoural cells and in non-tumoural tissues.

### Ethics statement

Mice were maintained in a specific pathogen-free animal facility, AniCan, with technical help of the Laboratoire des Modèles Tumoraux (LMT) and handled in accordance with the institutional guidelines and protocols approved by the animal care and use committee (Comité d'Evaluation Commun au Centre Léon Bérard, à l'Animalerie de transit de l'ENS, au PBES et au laboratoire P4; CECCAP).

### Chromatin immunoprecipitation assay

Doxorubicin-treated A549R cells were collected by enzymatic detachment. Cells were harvested in cold RBS (10 mM Tris pH 7,4, 10 mM NaCl, 5 mM MgCl2) added with protease inhibitors cocktail (PIC, Sigma–Aldrich, St. Louis, MO, USA) and cross-linked with 1.2% formaldehyde (CH_2_O, Sigma–Aldrich). After 10 min, cross-linking reaction was blocked by adding glycine to a final concentration of 125 mM, cells were incubated for 5 min on ice and washed three times with cold RBS plus PIC. Chromatin was shared by sonication (Sonifier 450, Branson, 3 mm microtip), centrifuged to pellet debris, and diluted 10 times in dilution buffer (50 mM Tris, pH 8.0, 0.5% NP-40, 0.2 M NaCl, 0.5 mM EDTA). Extracts (corresponding to about 2 × 10^6^ cells per condition) were precleared for 1 h with 80 µl of a 50% suspension of salmon sperm-saturated protein A (ss protein A). Immunoprecipitations were carried out at 4°C overnight using 2 µg p53 antibody (clone DO-1, SantaCruz Biotechnology). Immune complexes were collected with ss protein A (45 min) and washed three times (5 min each) with high salt buffer (washing buffer: 20 mM Tris, pH 8.0, 0.1% SDS, 1% NP-40, 2 mM EDTA, 500 mM NaCl) and three times with low salt buffer (1× Tris/EDTA [TE]). Immune complexes were extracted in 1× TE containing 1% SDS, and protein–DNA cross-links were reverted by heating at 65°C overnight. DNA was purified using QIAquick PCR Purification kit (Qiagen, Crawley, UK), according to the manufacturer's protocol. Quantitative real-time PCR analysis of regulatory sequences in input samples and immunoprecipitates was performed using the LightCycler FastStart DNA Master SYBR Green I Kit in LightCycler® 2.0 apparatus (Roche Applied Science).

### Netrin-1 gene reporter analysis

Cells (10^5^) were plated in 12-well plates and transfected with the different firefly luciferase reporters containing netrin-1 promoter constructs, described before (Delloye-Bourgeois et al, 2012; Paradisi et al, 2008), in presence or not of p53 expression vector. 24 h after transfection, cells were treated or not with 2 µM Doxorubicin and further incubated for 24 h. All transfections were performed in duplicate and the Dual-Luciferase Reporter Assay system (Promega, Charbonnieres, France) was carried out 48 h after transfection according to the manufacturer's protocol, using the Luminoskan Ascent apparatus (Thermolab System, Dreieich, Germany). As an internal control of transfection efficiency, the renilla luciferase encoding plasmid (pRL-CMV, Promega) was co-transfected and for each sample firefly luciferase activity was normalized to the renilla luciferase activity. Site-specific mutation of p53 binding site in netrin-1 promoter was performed using the QuickChange Site-directed Mutagenesis Kit (Stratagene) with mutagenic primers (GGACGGGGAGACCGGCTCTGCGATCCCCCTCGGGCGGC, sense and anti-sense), according to the manufacturer's protocol.

### Immunohistochemical staining

Immunohistochemistry was performed on 4 ovarian carcinomas specimen obtained at initial diagnosis and after chemotherapy. The blocks containing formalin-fixed paraffin embedded tumours were sectioned at a thickness of 4 µm. After deparaffinization and rehydratation, endogenous peroxidases were blocked by incubating the slides in 5% hydrogen peroxide in sterile water. Heat-induced treatment was performed with 10 mM high pH buffer (Dako, Trappes, France) in a PTlink for 30 min. The slides were then incubated with the goat polyclonal Netrin1 antibody (R&D Systems Lille, France) in the autostainer Dako (Trappes, France) for 60 min. The antibody was diluted using an antibody diluent solution (Chemmate, Dako) at 1/100. After rinsing in Phosphate Buffer Saline, the slides were incubated polyclonal rabbit anti-goat antibody (Dako) at 1/200 for 20 min and then with the Flex kit (Dako). Bound antibody was revealed by adding the substrate 3,3-diamino-benzidine. Sections were counterstained with haematoxylin.

### Statistical analysis

The data reported are the mean ± SD of at least three independent determinations, each performed in triplicate. Statistical analysis was performed by the nonparametric Mann–Whitney *U* test unless indicated.

### The paper explained

PROBLEM

Conventional chemotherapeutic agents such as Doxorubicin, 5-Fluorouracil, Paclitaxel (Taxol) and Cisplatin, represent the front line of cancer treatment in most cancers. However, their used are often associated with resistance and with toxicity. Netrin-1, a secreted factor upregulated in different cancer has become an interesting focus for targeted therapies. Biologics aiming to interfere with netrin-1/receptors interaction to trigger tumour cell death are under preclinical evaluation. Because clinical evaluation often assays the combination of a conventional treatment with a novel therapeutic strategy, we assessed the rationale of combining conventional treatment with biologics interfering with netrin-1/receptors interaction.

**RESULTS:**

We have observed that conventional treatments such as Doxorubicin, 5-Fluorouracil, Paclitaxel (Taxol) and Cisplatin treatments increased massively both netrin-1 and its receptors in various cancer cell lines and cancer specimen. Accordingly, we show that combining conventional treatments with biologics interfering with netrin-1/netrin-1 receptors interaction potentiates the effect of these conventional treatments both in cell culture and in mice model.

**IMPACT:**

This manuscript supports the view that combining conventional chemotherapies with netrin-1 interference could be a promising therapeutic approach.

## Author contributions

AP, MC, BG, GD, ER, DN, EC, IT, AB and PM designed the experiments. AP, MC, BG, GD, ER, DN and EC performed most of the experiments. IT performed immunostaining analysis. PC provided materials, CK, GN and PC provided useful insights. AP and PM wrote the manuscript.

## Notes

As a service to our authors and readers, this journal provides supporting information supplied by the authors. Such materials are peer reviewed and may be re-organized for online delivery, but are not copy-edited or typeset. Technical support issues arising from supporting information (other than missing files) should be addressed to the authors.
