# Supplementary material for: Combining chemotherapeutic agents and netrin-1 interference potentiates cancer cell death
Source: EMBO Mol Med. 2013 Oct 8;5(12):1821–34. doi: 10.1002/emmm.201302654 (PMC3914534; doi:10.1002/emmm.201302654)
Supplement: Supplementary file 1 [file emmm0005-1821-sd1.pdf]

EMBO Molecular Medicine

Combining chemotherapeutic agents and netrin-1 interference potentiates cancer cell death

Andrea Paradisi, Marion Creveaux, Benjamin Gibert, Guillaume Devailly, Emeline Redoulez, David Neves, Elsa Cleyssac, Isabelle Treilleux, Christian Klein, Gerhard Niederfellner, Philippe A. Cassier, Agnès Bernet and Patrick Mehlen

Corresponding author: Patrick Mehlen, CRCL

Review timeline:

Submission date:	19 February 2013
Editorial Decision:	09 April 2013
Revision received:	12 August 2013
Editorial Decision:	29 August 2013
Accepted:	06 September 2013

Transaction Report:

(Note: With the exception of the correction of typographical or spelling errors that could be a source of ambiguity, letters and reports are not edited. The original formatting of letters and referee reports may not be reflected in this compilation.)

Editor: Céline Carret

1st Editorial Decision 09 April 2013

Thank you for the submission of your manuscript to EMBO Molecular Medicine. We have now heard back from the three referees whom we asked to evaluate your manuscript. Although the referees find the study to be of potential interest, they also raise a number of concerns that we would like you to address in a revised version of this manuscript.

As you will see from the reports below, the reviewers suggest a certain number of experiments to improve the mechanistic insights of the study. Referee #2 also suggests demonstrating the specificity of the netrin1 system for cancer cells, and in agreement with referee #1, recommends testing p53 null and gain-of-function mutants, which we agree would be interesting.

As such, I would like to give you the opportunity to revise your manuscript, with the understanding that the referees' concerns must be fully addressed and that acceptance of the manuscript would entail a second round of review. Please note that that it is our journal's policy to allow only a single round of revision, and that acceptance or rejection of the manuscript will therefore depend on the completeness of your response and the satisfaction of the referees with it.

EMBO Molecular Medicine has a "scooping protection" policy, whereby similar findings that are published by others during review or revision are not a criterion for rejection. Should you decide to submit a revised version, I do ask that you get in touch after three months if you have not completed it, to update us on the status.

Please also contact us as soon as possible if similar work is published elsewhere. If other work is published we may not be able to extend the revision period beyond three months.

I look forward to receiving your revised manuscript.

***** Reviewer's comments *****

Referee #1 (Remarks):

Netrin-1 can act as a survival factor within the context of cancer, and its expression is elevated in diverse types of malignancies. Suppression of netrin expression or inhibition of its interaction with the receptors through which netrin acts can promote cell death and inhibit tumor growth. The present study tests the hypothesis that chemotherapeutic agents used in conjunction with netrin suppression may have efficacy against tumor cells and tumor growth. The notion that inhibition of the activities of a survival factor can be used against malignancies has been well studied. The present work applies this type of approach in another context and in detail.

The authors might comment on the similarities between oncogene addiction and the fact that some tumor cells seem to require netrin for survival. This is supported by the fact that chemotherapy induces netrin from some cells (Fig. 1), which can then use it to antagonize cell death.

After testing for modulation of netrin and netrin receptor expression in diverse cell lines (Fig. 2A), the authors test for these in ovarian cancer specimens (Fig. 2B). The authors should have a sentence or two here to justify testing in ovarian and not other cancers, to help the reader.

In figure 5, the authors test TRAP-netrin in conjunction with chemotherapy against tumors established from cells against which these agents were shown to work in cell culture. It would be more impressive if xenografts of human cancers were tested against these reagents. This would be a more real world test of the hypothesis. I would not insist on that here, but such xenografts are finding increased use. What is described here is a first step.

It would be interesting to see a time course for netrin expression in chemotherapy treated cells that are wild type for p53, p53 null, or express mutant p53. The putative p53-induced expression of netrin may be similar to that of p53-induction of Mdm2. The significance of this work would be supported by testing TRAP-netrin and chemotherapy against tumors from such types of cells. This type of test would be a first step in the eventual stratification of patients based on p53 genotype.

Referee #2 (Remarks):

This is an interesting manuscript focusing on the potential of various chemotherapeutics, operating through different mechanisms, to induce netrin1 and its dependence receptors, apparently selectively in cancer cells. The authors further explore this phenomenon to show that the observed induction of netrin 1 depends on wild-type p53 and specifically its binding to a motif in promoter B of netrin 1 gene. The upregulation provides protection against cell death, and interference with netrin1 interaction with its receptors potentiates the efficacy of chemotherapeutics, by inducing apoptosis both in vitro and in vivo. Overall, this is an interesting study relevant for the scope of the journal, with a potential to be exploited in the clinic. On the other hand, there are some issues (explained in detail below) that need to be properly addressed to strengthen the conclusions and the overall concept.

Major points

1. Novelty and generality - While the phenomenon of increased expression of netrin1 and its receptors in various subsets of tumors has been known, as was the dependency of upregulation of the receptors on p53, and also the strategy of interference with netrin1/receptor interaction to promote apoptosis was proposed and is being tested, there are some important novel observations in this study, which provide an advance in our understanding of this system. The two advances are: a) upregulation of netrin 1 and its receptors upon treatment with various drugs; and b) the p53 mediated regulation of netrin 1. One prediction from this concept is that insults which activate p53,

should also upregulate netrin1 and its receptors, and this generality would be nice to support by e.g. oxidative stress or some other 'non-chemotherapy' stress known to activate p53.

2. Another very important issue, and highly relevant to the success of this proposed treatment strategy, is the selectivity for cancer cells. In other words, when normal human epithelial cells and preferably also bone marrow (hematopoietic cells) in culture are exposed e.g. to doxorubicine, and p53 becomes activated, what happens to netrin 1 and its receptors, and apoptosis? This should be shown side by side with the cancer cell line used here, and if despite strong p53 activation the netrin1/receptor system is NOT upregulated, as presumed by this study, the authors should show whether this lack of response is due to the lack of p53 binding to netrin1 promoter, i.e. the mechanism they found in this study. This is critical to understand the selectivity and avoid normal tissue toxicity of the treatment in which combination of chemotherapy and netrin1 interference is used together, i.e. the main conclusion of this work.

3. The upregulation of dependence receptors is tested here only at the mRNA level, despite it is the protein level that matters in terms of the biological response. Therefore, at least one of the analyzed receptors should also be tested at the protein level in response to drugs, to see whether protein correlates with mRNA.

4. The increase of netrin 1 and its receptors after chemotherapy seen here in ovarian carcinomas is an important observation consistent with the proposed rationale for netrin1-interference treatment. What is however unclear is whether this increase represents an enrichment of netrin1-high cancer cells due to selection of already pre-treatment variant cancer cells that expressed higher level of netrin1 and hence were more resistant to treatment, or whether the tumor cell population as a whole is low in netrin 1 and after chemotherapy the expression of netrin 1 is 'uniformly' elevated/induced. This point is very relevant to the currently hotly debated issue on intratumor heterogeneity, and could easily be addressed by immunohistochemical staining of surgically treated tumors removed before any chemotherapy, to se whether there are any cancer cells with enhanced netrin 1/receptor levels prior to treatment.

5. The authors acknowledge that not only p53, but also other mechanisms such as NFkB can upregulate netrin1 and its receptors. This raises an important question with regard to cancer cell lines that harbor mutant p53, yet nevertheless induce netrin1/receptors after chemotherapy. The authors should examine whether in such a model, the upregulation is indeed dependent on NF-kB, since there could be yet another, alternative interpretation. Some p53 mutant proteins preserve some aberrant functions (known as gain-of-function) and since upregulation of netrin 1 is a protective mechanism that helps the tumor to survive and develop, it is feasible that some mutated forms of p53 can still upregulate netrin 1. This would then cause the observed lack of strict correlation between upregulation of netrin 1 after chemotherapy and the non-mutated state of p53, and it would represent another example of the gain-of-function role of mutant p53.

Other issues

6. It would be important to check the English language as presently there are many small errors throughout the manuscript, that should be corrected.

7. Also, while this may already be beyond this study, it would at least be interesting to discuss the possible resistance mechanisms of cancer cells exposed to the netrin-interference treatment. Are such mechanisms already known? E.g. the inability of receptors to trigger apoptosis despite the uncoupling from netrin 1? Or perhaps some redundancy of the ligands, some non-netrin1 ligand(s) that could still bind the receptors in the absence of netrin 1 binding, and thereby inhibit apoptosis?

Referee #3 (Comments on Novelty/Model System):

Technical quality - it is suggested to enhance the results of the qRT-PCR data with additional statistical tests of correlation.

Novelty - The mechanism is viewed as novel.

Impact - The MS focuses on issues of therapy resistance in a ligand-receptor system of interest in cancer biology.

Model system - A good diversity of cell lines was investigated.

Referee #3 (Remarks):

The manuscript describes p53-dependent induction of Netrin1 and receptors in response to chemotherapy. The topic is interesting as the ligand and its receptors are likely important determinants of carcinogenesis, although their function in chemotherapeutic response is less studied. A strength of the data is the analysis of the proposed mechanism in a panel of human cancer cell lines. It should be noted that in the abstract of a review by Arakawa (Nature Reviews Cancer, 2004), it is mentioned that p53 regulates Netrin-1 expression, but a personal communication is cited within the article and published data supporting this statement were not easily found. Overall it is viewed that the experimental approach and resulting data have the potential to provide a useful contribution in an important area of cancer biology.

Specific comments:

1) Figure 2A. Quantitative RT-PCR data should be reported as the numerical fold induction instead of the qualitative -, +, ++ approach. Correlations between Netrin expression and the following parameters should be reported: receptor expression, p53 status, drug IC50.

2) Figure 2A. The analysis of these results is too superficial and should be supported by greater analysis. Example: what does it mean that Panc-1 cells are p53-mutant, show no induction of Netrin1, but have a robust increase in DCC expression?

3) Figure 3. Controls demonstrating the efficacy of Netrin and Unc5B knockdown are needed.

4) Figure 4. Controls for the efficacy of the TRAP reagents are needed. Can a molecular readout of DCC/Unc5 receptor-induced cell death (perhaps receptor cleavage?) be measured +/- chemotherapeutic, +/- TRAP reagent?

5) Supplemental Figure 2E-H. The supplemental figure legends have not been transmitted to the reviewer. What is the meaning of the filled and empty symbols? What is the interpretation of SF2G?

1st Revision - authors' response 12 August 2013

Referee #1

"The authors might comment on the similarities between oncogene addiction and the fact that some tumor cells seem to require netrin for survival. This is supported by the fact that chemotherapy induces netrin from some cells (Fig. 1), which can then use it to antagonize cell death."

We thank the referee for his/her supportive comments. As suggested we now have discussed the similarities between oncogene addiction and netrin-1 up-regulation in the discussion section.

"After testing for modulation of netrin and netrin receptor expression in diverse cell lines (Fig. 2A), the authors test for these in ovarian cancer specimens (Fig. 2B). The authors should have a sentence or two here to justify testing in ovarian and not other cancers, to help the reader."

We have inserted a sentence justifying the analysis of ovarian cancer (one of the few cancer type available in our cancer in France where tumor biopsies are made before and after chemotherapy for the same patient).

"In figure 5, the authors test TRAP-netrin in conjunction with chemotherapy against tumors established from cells against which these agents were shown to work in cell culture. It would be more impressive if xenografts of human cancers were tested against these reagents. This would be a more real world test of the hypothesis. I would not insist on that here, but such xenografts are finding increased use. What is described here is a first step."

We agree with the referee that mice grafted with "fresh" human cancers are interesting alternative models to classic cell lines engrafted in nude mice. However they are also much more complicated to handle and to obtain a mice model bearing a human tumor that is not responsive to an anti-netrin-1 treatment, not (or slightly responsible to chemo alone) alone but is sensitive to both chemo+anti-netrin-1 treatment is not an easy task. The advantage of the cells engrafted is that we can test them in cell culture before testing in mice, even though we also agree that the use of a cell line engrafted model represents only a first step.

"It would be interesting to see a time course for netrin expression in chemotherapy treated cells that are wild type for p53, p53 null, or express mutant p53. The putative p53-induced expression of netrin may be similar to that of p53-induction of Mdm2. The significance of this work would be supported by testing TRAP-netrin and chemotherapy against tumors from such types of cells. This type of test would be a first step in the eventual stratification of patients based on p53 genotype."

As suggested, we have now included in Supplementary Figure 4 a time-course experiment with Doxorubicin, using p53-wild-type HCT116 cell line and its respective p53 null cells (Suppl.Fig. 4A). This strengthens the initial model as only the p53 wild-type cells showed a strong time-dependent induction of netrin-1 gene expression. We have also shown a parallel increase of p21 gene (Suppl.Fig.4B), a classical p53 target gene (p21 was used instead of Mdm2 gene suggested by the referee because in all the paper we used p21 gene as a control for p53 activation). Moreover, we also tested the effect of interfering with netrin-1/receptors interaction and Doxorubicin treatment in these cell lines. As shown in Suppl.Fig.4C, only p53 wild-type cells, showing netrin-1 up-regulation following chemotherapeutic treatment, are sensitive to the combined treatment.

Referee #2

"This is an interesting manuscript focusing on the potential of various chemotherapeutics, operating through different mechanisms, to induce netrin1 and its dependence receptors, apparently selectively in cancer cells. The authors further explore this phenomenon to show that the observed induction of netrin 1 depends on wild-type p53 and specifically its binding to a motif in promoter B of netrin 1 gene. The upregulation provides protection against cell death, and interference with netrin1 interaction with its receptors potentiates the efficacy of chemotherapeutics, by inducing apoptosis both in vitro and in vivo. Overall, this is an interesting study relevant for the scope of the journal, with a potential to be exploited in the clinic."

We thank the referee for his/her kind comments.

"Novelty and generality - While the phenomenon of increased expression of netrin1 and its receptors in various subsets of tumors has been known, as was the dependency of upregulation of the receptors on p53, and also the strategy of interference with netrin1/receptor interaction to promote apoptosis was proposed and is being tested, there are some important novel observations in this study, which provide an advance in our understanding of this system. The two advances are: a) upregulation of netrin 1 and its receptors upon treatment with various drugs; and b) the p53 mediated regulation of netrin 1. One prediction from this concept is that insults which activate p53, should also upregulate netrin1 and its receptors, and this generality would be nice to support by e.g. oxidative stress or some other 'non-chemotherapy' stress known to activate p53."

As suggested, we now added in Supplementary Figure 4 the effect of oxidative stress to netrin-1 expression. Treatment of A549R cells with hydrogen peroxide (H_2O_2) induced netrin-1 up-regulation, as well as the over-expression of the p53 target gene p21 (Suppl.Fig.4EF), supporting the interesting point made by the referee.

"Another very important issue, and highly relevant to the success of this proposed treatment strategy, is the selectivity for cancer cells. In other words, when normal human epithelial cells and preferably also bone marrow (hematopoietic cells) in culture are exposed e.g. to doxorubicine, and p53 becomes activated, what happens to netrin 1 and its receptors, and apoptosis? This should be shown side by side with the cancer cell line used here, and if despite strong p53 activation the netrin1/receptor system is NOT upregulated, as presumed by this study, the authors should show whether this lack of response is due to the lack of p53 binding to netrin1 promoter, i.e. the mechanism they found in this study. This is critical to understand the selectivity and avoid normal tissue toxicity of the treatment in which combination of chemotherapy and netrin1 interference is used together, i.e. the main conclusion of this work."

The selectivity is indeed of major importance. As initially shown in Fig.5D, when mice where treated with doxorubicin, we failed to detect increase of netrin-1 expression in most tissues tested. This is also exemplified now by looking at netrin-1 expression before and after chemotherapies in human patient by netrin-1 immunohistochemistry (Fig.2C) where it can be seen that netrin-1 is clearly up-regulated in tumor cells while this up-regulation is not detected in the adjacent "normal" cells. However the selectivity is not complete as we have seen in vitro in the Weinberg HMEC/HTERT model, that doxorubicin treatment actually up-regulates netrin-1 in both HMEC and HTERT model.

"The upregulation of dependence receptors is tested here only at the mRNA level, despite it is the protein level that matters in terms of the biological response. Therefore, at least one of the analyzed receptors should also be tested at the protein level in response to drugs, to see whether protein correlates with mRNA."

We agree with the referee that up-regulation of the receptor itself was missing. To combine expression of the protein and the actual membrane localization of the receptor, we thus analyzed expression of DCC following Doxorubicin treatment by flow cytometry analysis of DCC (Suppl.Fig.1A). This now strengthen a correlation between DCC mRNA level up-regulation and increase receptor expression at the plasma membrane.

"The increase of netrin 1 and its receptors after chemotherapy seen here in ovarian carcinomas is an important observation consistent with the proposed rationale for netrin1-interference treatment. What is however unclear is whether this increase represents an enrichment of netrin1-high cancer cells due to selection of already pre-treatment variant cancer cells that expressed higher level of netrin1 and hence were more resistant to treatment, or whether the tumor cell population as a whole is low in netrin 1 and after chemotherapy the expression of netrin 1 is 'uniformly' elevated/induced. This point is very relevant to the currently hotly debated issue on intratumor heterogeneity, and could easily be addressed by immunohistochemical staining of surgically treated tumors removed before any chemotherapy, to se whether there are any cancer cells with enhanced netrin 1/receptor levels prior to treatment."

As suggested, we performed an immunohistological staining on tumors obtained before and after chemotherapy (Fig.2C). In three of the four patients analyzed, we have found a general low netrin-1 staining in biopsies obtained at the diagnosis, and an intermediate-high netrin-1 staining after chemotherapy. These results suggest indeed that netrin-1 is expressed at low levels in these tumors, and that chemotherapy induces an elevated and uniform netrin-1 expression.

"The authors acknowledge that not only p53, but also other mechanisms such as NFkB can upregulate netrin1 and its receptors. This raises an important question with regard to cancer cell

lines that harbor mutant p53, yet nevertheless induce netrin1/receptors after chemotherapy. The authors should examine whether in such a model, the upregulation is indeed dependent on NF-kB, since there could be yet another, alternative interpretation. Some p53 mutant proteins preserve some aberrant functions (known as gain-of-function) and since upregulation of netrin 1 is a protective mechanism that helps the tumor to survive and develop, it is feasible that some mutated forms of p53 can still upregulate netrin 1. This would then cause the observed lack of strict correlation between upregulation of netrin 1 after chemotherapy and the non-mutated state of p53, and it would represent another example of the gain-of-function role of mutant p53."

A deepened analysis of netrin-1 up-regulation in cell lines after chemotherapy showed a more strict correlation between netrin-1 over-expression and p53 state. Indeed, only two cell lines harboring mutated p53 (MiaPacA and PA1) showed a modest increase of netrin-1 expression (Fig.2A), while p53-wild-type cell lines presented a more robust netrin-1 up-regulation, often with two or more chemotherapeutic drugs known to activate p53 (Cisplatin, Doxorubicin and Taxol). As noted by the referee, we have shown that netrin-1 is an NF-kB target gene (Paradisi et al, Gastroenterology, 2008), and this transcription factor is known to be sometimes involved in chemoresistance. However, in different cell line tested we failed to inhibit doxorubicin-induced netrin-1 up-regulation by inhibiting NFkB activity (by silencing the major subunit of NF-kB, the protein p65 (data not shown)). Yet, it is fair to say that even though in the cell lines tested here we haven't shown the implication of NFkB, it may actually occurs in other p53 mutant cell line or in real in vivo condition. We also agree with the referee that some gain-of-function mutants of p53 could positively regulate netrin-1 expression. However, by testing a series of classic p53mutants, we have failed to find such positive regulation. As shown in Suppl.Fig.4D, over-expression of seven p53 mutants did not affect netrin-1 promoter activity.

"It would be important to check the English language as presently there are many small errors throughout the manuscript, that should be corrected."

We have carefully checked English in the text and corrected small errors.

"Also, while this may already be beyond this study, it would at least be interesting to discuss the possible resistance mechanisms of cancer cells exposed to the netrin-interference treatment. Are such mechanisms already known? E.g. the inability of receptors to trigger apoptosis despite the uncoupling from netrin 1? Or perhaps some redundancy of the ligands, some non-netrin1 ligand(s) that could still bind the receptors in the absence of netrin 1 binding, and thereby inhibit apoptosis?"

The mechanism of resistance to netrin-interference treatment have not been described so far but as suggested by the referee we indeed agree that such resistance is expected either by inactivation of the receptor or of the downstream apoptotic pathway.

Referee #3

"The manuscript describes p53-dependent induction of Netrin1 and receptors in response to chemotherapy. The topic is interesting as the ligand and its receptors are likely important determinants of carcinogenesis, although their function in chemotherapeutic response is less studied. A strength of the data is the analysis of the proposed mechanism in a panel of human cancer cell lines. It should be noted that in the abstract of a review by Arakawa (Nature Reviews Cancer, 2004), it is mentioned that p53 regulates Netrin-1 expression, but a personal communication is cited within the article and published data supporting this statement were not easily found. Overall it is viewed that the experimental approach and resulting data have the potential to provide a useful contribution in an important area of cancer biology"

We thank the referee for his/her kind comment.

"Technical quality - it is suggested to enhance the results of the qRT-PCR data with additional statistical tests of correlation."

As suggested by the referee, we re-analyzed the qRT-PCR data of netrin-1 and its receptors expression in cell lines and we performed statistical analysis of gene expression variations.

"Figure 2A. Quantitative RT-PCR data should be reported as the numerical fold induction instead of the qualitative -, +, ++ approach. Correlations between Netrin expression and the following parameters should be reported: receptor expression, p53 status, drug IC50."

qRT-PCR data in Figure 2A are now reported as the numerical fold induction. p53 status for all the cell lines tested is now represented (2nd column), as well as a correlation with drug resistance (gray box for drug resistant cell lines). However, for clarity, we decided to not include the data of receptor expression in the same table of netrin-1.

"Figure 2A. The analysis of these results is too superficial and should be supported by greater analysis. Example: what does it mean that Panc-1 cells are p53-mutant, show no induction of Netrin1, but have a robust increase in DCC expression?"

We agree with the referee that the analysis and discussion was incomplete. Regarding the specific point on DCC, we actually have no clue on whether DCC up-regulation in the several cell lines tested following chemotherapeutic agents treatment is correlated with p53 activation. Indeed, while UNC5 receptors are described as p53 target genes, we have only very limited (if none) information regarding DCC expression regulation.

"Figure 3. Controls demonstrating the efficacy of Netrin and Unc5B knockdown are needed."

Efficacy of netrin-1 and UNC5B knockdown is now added in Supplementary Figure 2A.

"Figure 4. Controls for the efficacy of the TRAP reagents are needed. Can a molecular readout of DCC/Unc5 receptor-induced cell death (perhaps receptor cleavage?) be measured +/- chemotherapeutic, +/- TRAP reagent?"

As suggested, we added in Supplementary Figure 2C the analysis of the dephosphorylation of DAPK in presence of both TRAP-netrin reagent and Doxorubicin, a molecular event associated with cell death induced by unbound UNC5B receptor (Llambi et al, EMBO J, 2005).

"Supplemental Figure 2E-H. The supplemental figure legends have not been transmitted to the reviewer. What is the meaning of the filled and empty symbols? What is the interpretation of SF2G?"

This was indeed an uploading error from our side. Supplemental Figure legends are now transmitted to the referees.

2nd Editorial Decision 29 August 2013

Thank you for the submission of your revised manuscript to EMBO Molecular Medicine. We have now received the enclosed reports from the referees that were asked to re-assess it. As you will see the reviewers are now supportive and I am pleased to inform you that we will be able to accept your manuscript pending the following final amendments:

-Please provide an ethic statement regarding the use of mice as described in our guidelines

-We now encourage the publication of source data, particularly for electrophoretic gels and blots, with the aim of making primary data more accessible and transparent to the reader. Would you be willing to provide a single PDF file comprising the original, uncropped and unprocessed scans of all

or key gels used in the figures? These should be labeled with the appropriate figure/panel number, and should have molecular weight markers; further annotation could be useful but is not essential. This PDF will be published online with the article as a supplementary "Source Data" file. If you have any questions regarding this just contact me.

-Figures 1D, 2C and 6A, please provide a size bar for the micrographs.

Please submit your revised manuscript within two weeks.

I look forward to receiving a new revised version of your manuscript.

***** Reviewer's comments *****

Referee #1 (Comments on Novelty/Model System):

This is a timely study that has been well executed experimentally.

Referee #1 (Remarks):

This is a scholarly piece of work and the authors are to be congratulated.

Referee #3 (Remarks):

The manuscript appears to have been significantly strengthened in the revision, which addressed all of my previous concerns.
