# Supplementary material for: Combining chemotherapeutic agents and netrin-1 interference potentiates cancer cell death
Source: EMBO Mol Med. 2013 Oct 8;5(12):1821–34. doi: 10.1002/emmm.201302654 (PMC3914534; doi:10.1002/emmm.201302654)
Supplement: Supplementary file 2 [file emmm0005-1821-sd2.pdf]

Combining chemotherapeutic agents and netrin-1 interference potentiates cancer cell death.

Andrea Paradisi et al.

Supplementary Figure 1, 2, 3 and 4
Supplementary Table 1

Supplementary Figure Legends

Supplementary Figure 1: Netrin-1 receptors gene expression following cytotoxic drugs treatment.

(A): Flow cytometry analysis of DCC protein expression following Doxorubicin treatment. A549 cells were treated for 48 hours with 2*μ*M Doxorubicin, and freshly incubated with α-DCC antibody. The fluorescence shift in Doxorubicin-treated cells (DoxoR, blue), compared to control, not-treated cells (NT, red) indicated an increase of extracellular DCC receptor. The green (DoxoR-DCC) flow indicates the fluorescence profile of Doxorubicin-treated A549 cells, incubated with a control antibody. **(B):** Cancer cells were treated as described in Fig.2A, and UNC5A and UNC5C gene expression was evaluated after drugs treatment. Scoring system is the same used in Fig.2A. Both receptors showed poor expression levels changes after treatment, as compared to untreated cells. **(C,D,E,F):** Netrin-1 receptors expression levels in ovarian biopsies of tumors from patients before and after carboplatin/taxol treatment. The median values were calculated from each group. UNC5B (B), UNC5C (C), UNC5D (D) and DCC (E) gene expression showed a similar up-regulation after chemo-therapeutic treatment. Gene expression levels were normalized to Glyceraldehyde 3-phosphate dehydrogenase (GAPDH), used as housekeeping gene. Mann–Whitney tests were performed, and *P* values are indicated.

Supplementary Figure 2: Validation of netrin-1/receptors interference.

(A,B): Netrin-1 (NTN1) and UNC5B expression in siRNA-transfected A549R cells. Cells were transfected with netrin-1 (siNet) or UNC5B (siUNC5B) siRNA alone or in combination, as described in Fig.2. The efficiency of gene silencing in control or Doxorubicin-treated cells was measured by quantitative PCR. Netrin-1 and UNC5B gene expression was normalized with GAPDH. **(C):** Doxorubicin and netrin-1 interfering

protein treatment induces dephosphorylation of DAPK. A549 cells were treated for 48 hours with Doxorubicin in presence or not of 2µg/mL TRAP-netrin^{UNC5A}, and the levels of phosphorylated DAPK was evaluated by western blotting. While Doxorubicin and TRAP-netrin^{UNC5A} alone did not affect DAPK phosphorylation, combined treatment induced a strong dephosphorylation of DAPK.

Supplementary Figure 3: Doxorubicin triggers p53 activation.

(A): A549 cells were treated with the indicated doses of Doxorubicin for 48 hours, and p53 was detected by western blot in the protein lysate, using the α-p53-DO-1 antibody. β-actin was used as housekeeping gene to normalize protein content. **(B):** Endogenous p53 (green) accumulation and nuclear translocation following treatment with 2*µ*M Doxorubicine for 48 hours was confirmed by immunofluorescence staining. Nuclei were counterstained using Hoescht staining (blue). **(C):** p21 gene expression in A549 cells, treated with the indicated doses of Doxorubicin for 24 hours, was evaluated by quantitative PCR and normalized to the housekeeping gene (GAPDH). The amount of p21 mRNA, relative to untreated cells, was calculated using the comparative C_T method. **(D):** Accumulation of p21 protein following Doxorubicin treatment was confirmed by western blot, using the α-p21-C-19 antibody. **(E,F,G,):** Correlation between p53 target genes and netrin-1 gene expression in ovarian tumor biopsies from patients before and after a chemotherapeutic cycle of carboplatin/taxol treatment. Expression of p21 (**E**) and GADD45 (**F**) genes, known to be up-regulated by p53 activation, was plotted in function of netrin-1 (NTN1) gene expression. To quantify the goodness of fit, the coefficient of determination R^2 was calculated. As a control, the expression of the netrin-1 receptor UNC5A, which was not affected by chemotherapeutic treatment in these samples, was plotted *versus* the netrin-1 expression. Results showed that while the p53 target genes significantly correlated with netrin-1 expression, the UNC5A gene expression did not fit with netrin-1 gene expression. Filled rounds represent sample tumors obtained from

patients after chemotherapeutic treatment, while empty round correspond to sample tumors from patients before treatment.

Supplementary Figure 4: Wild-type p53 is required for netrin-1 up-regulation.

(A,B): p53-wild-type HCT116 (+/+) or p53-knock-out (KO) HCT116 (-/-) cell lines were treated with 2μM Doxorubicin and cells were collected at the indicated times. Netrin-1 (NTN1, A) and p21 (B) gene expression levels, normalized with Glyceraldehyde 3-phosphate dehydrogenase (GAPDH) and expressed as fold over control, were then evaluated by quantitative PCR.. **(C):** p53- and KO-HCT116 cells were treated for 48 hours with increasing concentrations of Doxorubicin, in presence or not of 2µg/mL TRAP-netrin^{UNC5A} recombinant protein, and cell survival was measured by MTS assay and normalized to untreated cells. While TRAP-netrin^{UNC5A} treatment sensitizes p53-wild-type HCT116 cells to Doxorubicin treatment, depletion of p53 is sufficient to restore Doxorubicin resistance also in presence of the netrin-1 interfering protein. **(D):** p53-KO HCT116 cells were transfected with the NetPB promoter, containing the p53 binding site, in the presence of empty vector (pcDNA), p53 wild-type construct (WT) or the indicated p53 mutants constructs. 24 hours after transfection, promoter activity was measured by luciferase assay. Expression of p53 constructs was analyzed by western blot. **(E,F):** p53-wild-type HCT116 cells were treated with 500μM hydrogen peroxide (H_2O_2) for 24 hours. After RNA extraction, netrin-1 and p21 gene expression levels, normalized with GAPDH gene, were evaluated. H_2O_2 is able to activate p53, as indicated by the induction of p21 (F), and to induce netrin-1 gene expression (E). *, $P<0.05$; **, $P<0.01$; ***, $P<0.001$; DoxoR, Doxorubicin.

Supplementary Table 1: Cell sensitivity to cytotoxic drugs.

The inhibitory concentration (IC) IC_{10}, IC_{30} and IC_{50} in response to Cisplatin, 5-Fluoruracil (5FU), Doxorubicin, and paclitaxel (Taxol) was determined for the indicated

cell lines by MTS assays. IC_{50} values were calculated by linear regression of double reciprocal plots. For resistant cancer cell lines (i.e., more than 50% cell survival after treatment with maximal drugs concentrations IC_{MAX}), represented by gray boxes, IC_{MAX} and fractions were calculated.

A

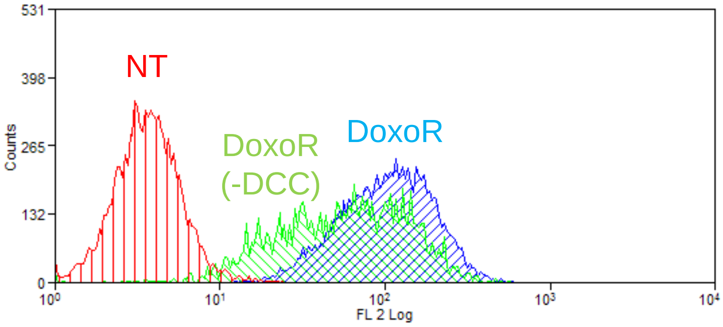

B

UNC5A				
**Cell line**	**Cisplatin**	**5-Fluorouracil**	**Doxorubicin**	**Taxol**
HBL100	-	-	-	-
A549R	-	-	45 ± 10*	-
H322	- (ne)	- (ne)	- (ne)	- (ne)
H358	- (ne)	- (ne)	- (ne)	- (ne)
HCT116	-	-	-	-
HCT8	- (ne)	- (ne)	- (ne)	- (ne)
SH-Sy5Y	-	-	-	-
IMR32	-	-	-	-
U87MG	- (ne)	- (ne)	- (ne)	- (ne)
SF767	- (ne)	- (ne)	- (ne)	- (ne)
MiaPacA	21.6 ± 4.9*	4.6 ± 1.4*	43.0 ± 0.4***	16.6 ± 4.5*
Panc-1	- (ne)	- (ne)	- (ne)	- (ne)
PA-1	- (ne)	- (ne)	- (ne)	- (ne)
TOV-112D	-	-	-	-
NIH-OVCAR3	-	-	8.0 ± 0.5**	2.1 ± 0.1**
Positive cells (%)	1/15	1/15	3/15	2/15
	**7%**	**7%**	**20%**	**13%**

UNC5C				
**Cell line**	**Cisplatin**	**5-Fluorouracil**	**Doxorubicin**	**Taxol**
HBL100	-	-	-	-
A549R	-	-	-	-
H322	-	-	3.4 ± 0.1***	-
H358	-	-	5.0 ± 1.0*	-
HCT116	-	-	16.9 ± 0.5***	-
HCT8	- (ne)	- (ne)	- (ne)	- (ne)
SH-Sy5Y	-	-	-	-
IMR32	-	-	-	-
U87MG	-	-	-	-
SF767	-	-	146 ± 33*	-
MiaPacA	-	-	-	-
Panc-1	-	-	-	-
PA-1	- (ne)	- (ne)	- (ne)	- (ne)
TOV-112D	-	-	-	-
NIH-OVCAR3	-	-	-	-
Positive cells (%)	0/15	0/15	4/15	0/15
	**0%**	**0%**	**27%**	**0%**

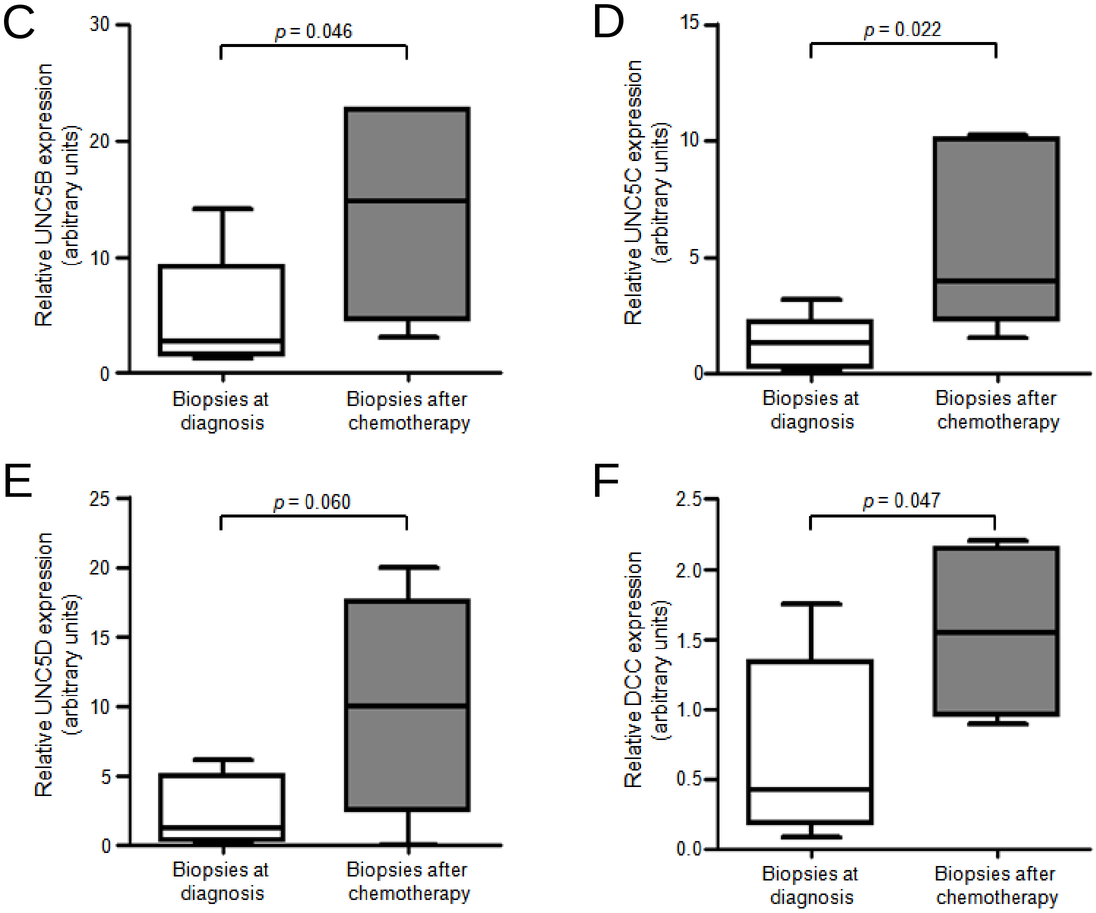

Supplementary Figure 2

A

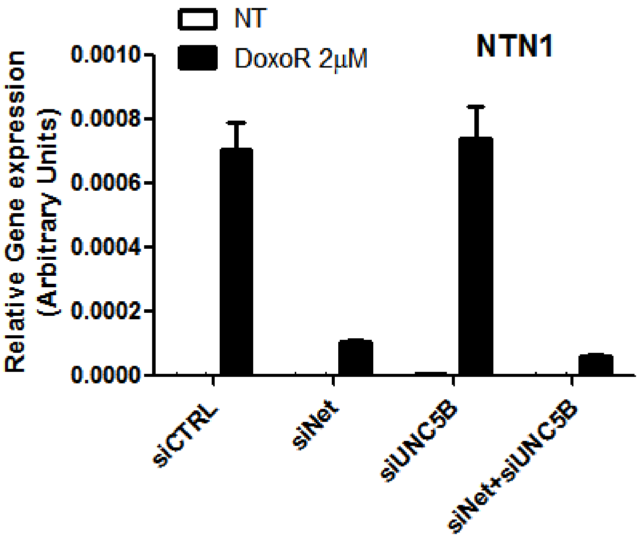

B

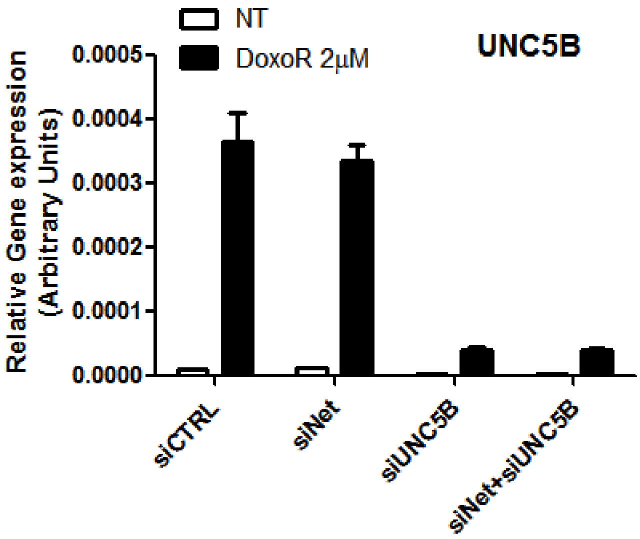

C

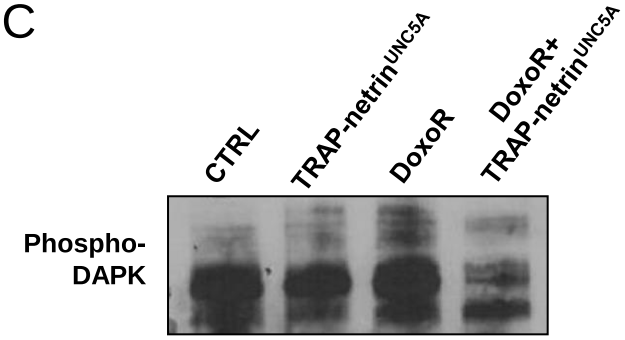

Supplementary Figure 3

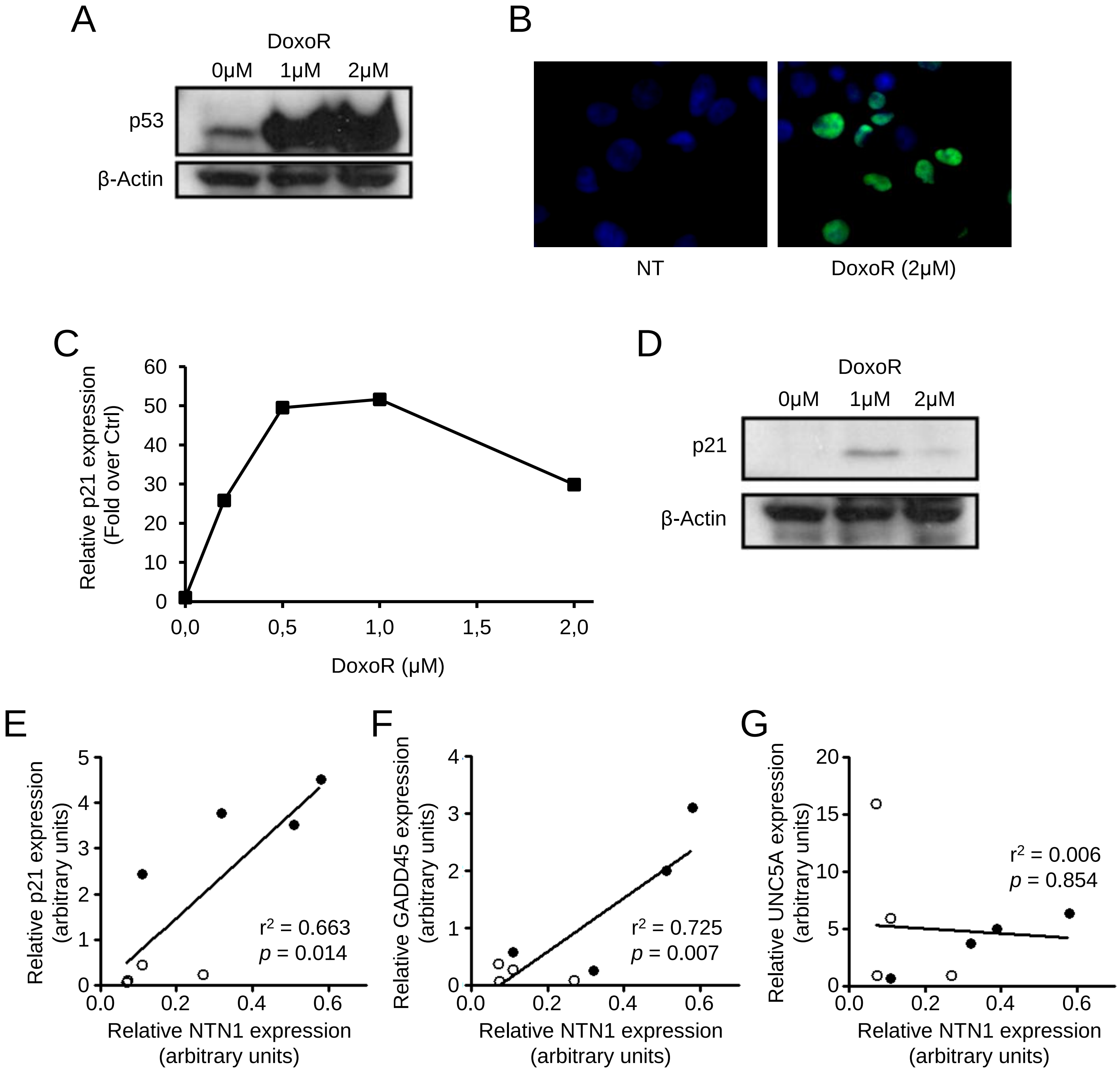

Supplementary Figure 4

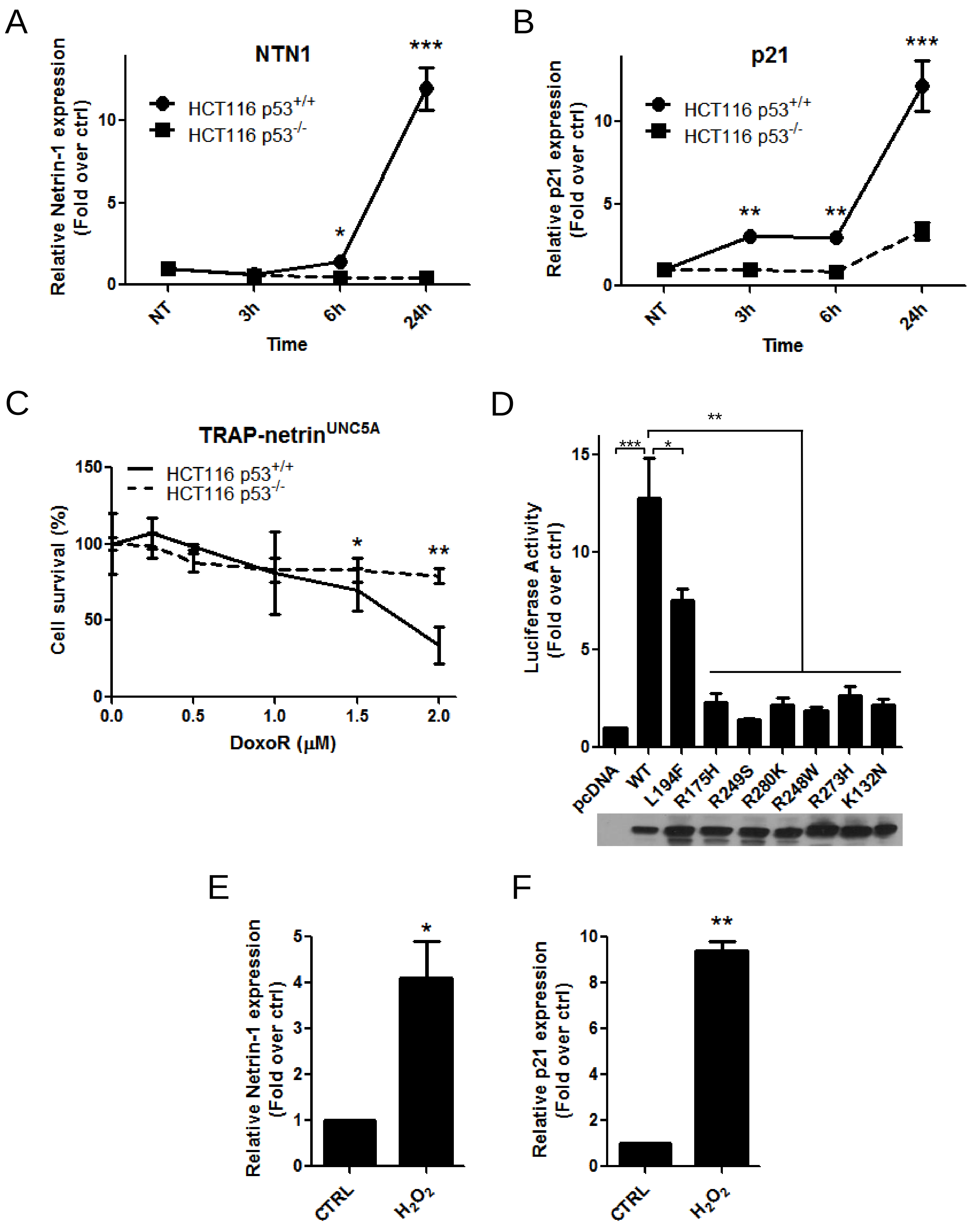

Supplementary Table 1

	Cisplatin			5-Fluorouracil			Doxorubicin			Taxol		
	IC_{10}	IC_{30}	IC_{50}	IC_{10}	IC_{30}	IC_{50}	IC_{10}	IC_{30}	IC_{50}	IC_{10}	IC_{30}	IC_{50}
HBL100	0.5μM	1.5μM	2.5μM	0.036μM	0.108μM	0.18μM	0.04μM	0.12μM	0.2μM	0.6nM	1.8nM	3nM
A549R	10μM[1]	25μM[2]	50μM[3]	0.4μM[1]	1μM[2]	2μM[3]	1μM[1]	2.5μM[2]	5μM[3]	100nM[1]	250nM[2]	500nM[3]
H322	10μM[1]	25μM[2]	50μM[3]	0.4μM[1]	1μM[2]	2μM[3]	0.36μM	1.08μM	1.8μM	1nM	3nM	5nM
H358	0.48μM	1.44μM	2.4μM	0.4μM[1]	1μM[2]	2μM[3]	0.15μM	0.45μM	0.75μM	1nM	3nM	5nM
HCT116	7μM	21μM	35μM	4μM[1]	10μM[2]	20μM[3]	1μM[1]	2.5μM[2]	5μM[3]	0.08nM	0.24nM	0.4nM
HCT8	1.2μM	3.6μM	6μM	0.25μM	0.75μM	1.25μM	0.36μM	1.08μM	1.8μM	4nM	12nM	20nM
SH-Sy5Y	0.35μM	1.05μM	1.75μM	0.03μM	0.09μM	0.15μM	1μM[1]	2.5μM[2]	5μM[3]	100nM[1]	250nM[2]	500nM[3]
IMR32	0.4μM	1.2μM	2μM	0.4μM[1]	1μM[2]	2μM[3]	0.2μM	0.6μM	1μM	0.3nM	0.9nM	1.5nM
U87MG	1.24μM	3.72μM	6.2μM	0.25μM	0.75μM	1.25μM	0.2μM	0.6μM	1μM	2nM	6nM	10nM
SF767	0.4μM	1.2μM	2μM	0.46μM	1.38μM	2.3μM	0.24μM	0.72μM	1.2μM	1nM	3nM	5nM
MiaPacA	10μM[1]	25μM[2]	50μM[3]	0.4μM[1]	1μM[2]	2μM[3]	0.76μM	2.28μM	3.8μM	1nM	3nM	5nM
Panc-1	10μM[1]	25μM[2]	50μM[3]	0.4μM[1]	1μM[2]	2μM[3]	1μM[1]	2.5μM[2]	5μM[3]	100nM[1]	250nM[2]	500nM[3]
PA-1	0.12μM	0.36μM	0.6μM	0.6μM	1.8μM	3μM	0.16μM	0.48μM	0.8μM	0.8nM	2.4nM	4nM
TOV-112D	1.46μM	4.4μM	7.3μM	4.88μM	14.64μM	24.4μM	0.34μM	1.02μM	1.7μM	10.4nM	31.1nM	51.9nM
NIH-OVCAR3	2.8μM	8.5μM	14.1μM	1.72μM	5.16μM	8.6μM	0.68μM	2.04μM	3.4μM	6.3nM	18.9nM	31.5nM

[1], 1/5 IC_{MAX} ; [2], 1/2 IC_{MAX}; [3], IC_{MAX}
