# Supplementary figures and images for: Combining chemotherapeutic agents and netrin-1 interference potentiates cancer cell death
Source: EMBO Mol Med. 2013 Oct 8;5(12):1821–34. doi: 10.1002/emmm.201302654 (PMC3914534; doi:10.1002/emmm.201302654)

Figure 1C

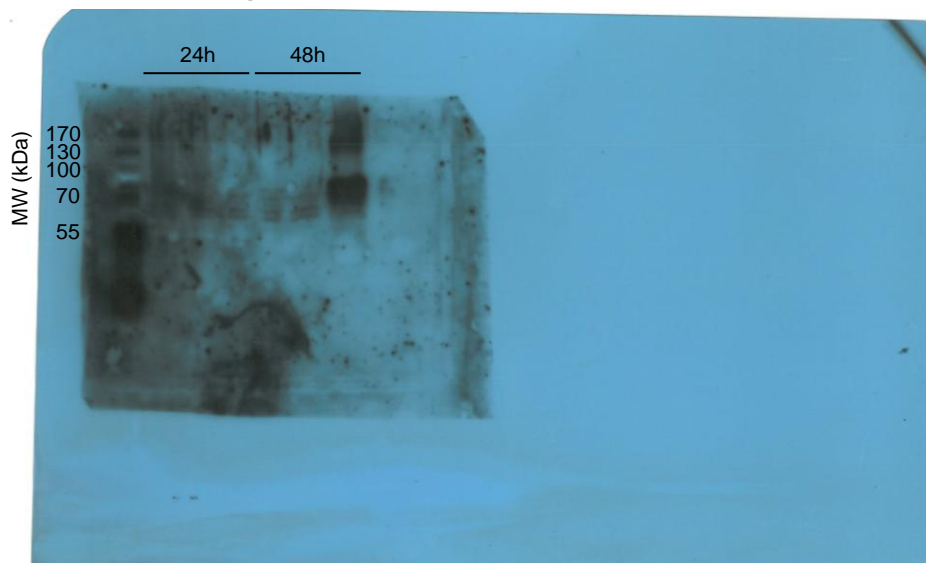

Supplementary Figure 3A/3D

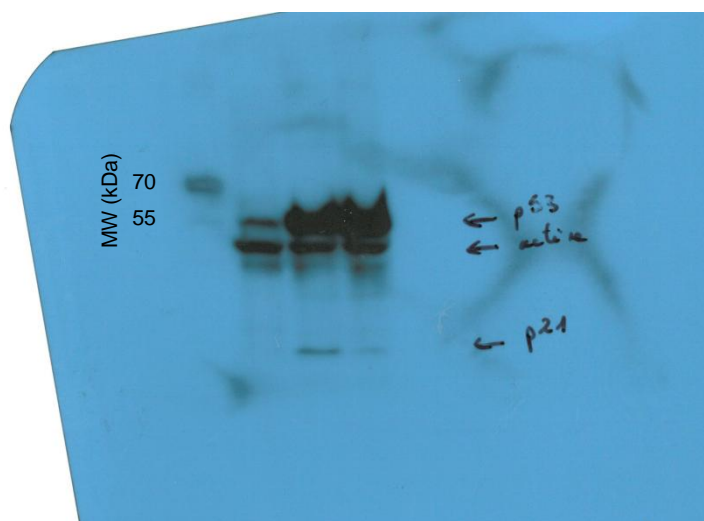

Supplementary Figure 2C

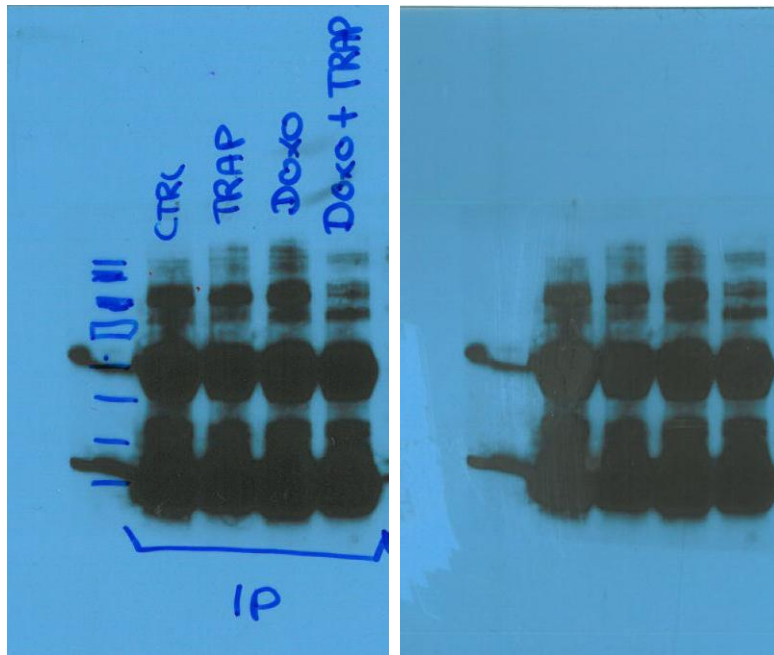

Supplementary Figure 4D

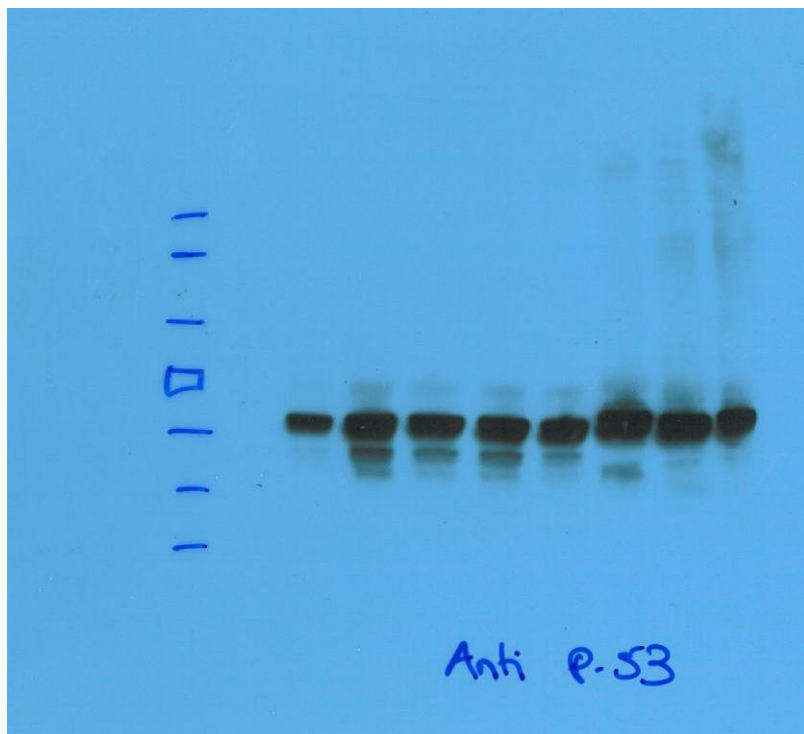

Supplement: Supplementary file 3 [file emmm0005-1821-sd3.pdf]
